# DNA damage enhances integration of HIV-1 into macrophages by overcoming integrase inhibition

**DOI:** 10.1186/1742-4690-10-21

**Published:** 2013-02-21

**Authors:** Takayoshi Koyama, Binlian Sun, Kenzo Tokunaga, Masashi Tatsumi, Yukihito Ishizaka

**Affiliations:** 1 Department of Intractable Diseases, National Center for Global Health and Medicine, 1-21-1 Toyama, 162-8655, Shinjuku-ku, Tokyo, Japan; 2 Research Group of HIV Molecular Epidemiology and Virology, The State Key Laboratory of Virology, Wuhan Institution of Virology, Chinese Academy of Sciences, 430071, Wuhan, Hubei, China; 3 Department of Pathology, National Institute of Infectious Diseases, 1-23-1 Toyama, 162-8640, Shinjuku-ku, Tokyo, Japan; 4 AIDS Research Center, National Institute of Infectious Diseases, 1-23-1 Toyama, 162-8640, Shinjuku-ku, Tokyo, Japan

**Keywords:** DNA damage, HIV-1, Integrase inhibitor, Integration, Resting macrophages, Vpr

## Abstract

**Background:**

The prevention of persistent human immunodeficiency virus type 1 (HIV-1) infection requires the clarification of the mode of viral transduction into resting macrophages. Recently, DNA double-strand breaks (DSBs) were shown to enhance infection by D64A virus, which has a defective integrase catalytic activity (IN-CA). However, the mechanism by which DSBs upregulate viral transduction was unclear. Here we analyzed the roles of DSBs during IN-CA–independent viral transduction into macrophages.

**Results:**

We used cellular systems with rare-cutting endonucleases and found that D64A virus integrated efficiently into the sites of artificially induced DSBs. This IN-CA-independent viral transduction was blocked by an inhibitor of ataxia telangiectasia mutated protein (ATM) but was resistant to raltegravir (RAL), an inhibitor of integrase activity during strand transfer. Moreover, Vpr, an accessory gene product of HIV-1, induced DSBs in resting macrophages and significantly enhanced the rate of IN-CA-independent viral transduction into macrophages with concomitant production of secondary viruses.

**Conclusion:**

DSBs contribute to the IN-CA–independent viral infection of macrophages, which is resistant to RAL. Thus, the ATM-dependent cellular pathway and Vpr-induced DNA damage are novel targets for preventing persistent HIV-1 infection.

## Background

The prognosis of individuals infected with human immunodeficiency virus type 1 (HIV-1) has improved due to the development of combination antiretroviral therapy (cART) [1]. However, several lines of evidence revealed that the current regimen does not block viral replication completely [2], which promotes the emergence of drug-resistant mutant viruses. Recently, new anti-retroviral drugs that target viral entry or the integration of viral DNA into the host genome have been applied clinically [3,4], which allows the possibility of overcoming viruses that are resistant to conventional cART. Moreover, an advanced study directed at the development of novel anti-HIV-1 compounds attempted to identify the cellular proteins that associate with HIV-1 proteins [5]. Macrophages are less sensitive to the toxic effects of HIV-1 and they function as persistent producers of the virus [2]; therefore, it is important to develop novel anti-HIV-1 compounds that target viral transduction into resting macrophages.

Integrase, a 288-amino-acid and 32-kDa HIV-1 protein, promotes strand-transfer reaction [6], where the reverse-transcribed double-stranded viral DNA is integrated into the host genome. The integrase catalytic activity (IN-CA) excises two nucleotides from the 3^′^-end of the viral DNA and the CA-3^′^-OH is ligated to the 5^′^-*O*-phosphate end of the genomic DNA [6]. All these strand transfer steps depend on the presence of a D,D(35)E motif in the central domain and any mutations in this motif abrogate the activity required for the strand-transfer process [7]. Notably, single-strand gaps are produced in both regions flanking the viral DNA and it was postulated that cellular factors repair these gaps because viral proteins have a low DNA damage repair activity [8].

Initially, Daniel *et al.* proposed that DNA-dependent protein kinase was a cellular factor involved in gap-repair [9], and then ataxia telangiectasia mutated (ATM), ataxia telangiectasia and Rad3-related (ATR), Nijmegen breakage syndrome 1 (NBS1), and poly(ADP-ribose) polymerase 1 (PARP1) have also been nominated as cellular proteins involved in efficient viral transduction [10-13]. Using KU55933, a specific ATM inhibitor, Lau *et al.* proposed that ATM is also involved in HIV-1 transduction [14], whereas Sakurai *et al.* demonstrated that DNA damage repair enzymes are involved in multiple steps of retroviral infection [15]. These observations support the importance of DNA double-strand breaks (DSBs) in viral transduction, although their roles are controversial [16-19]. A possible explanation for discrepancies in reported observations is that the single-strand gaps are repaired in a redundant fashion by DNA damage repair enzymes, the expression of which varies among cells [20]. It is also possible that DSBs have modest effects on viral transduction, which may be overwhelmed by the infectivity of the wild-type (WT) virus. This suggests that it is important to evaluate the effects of DSBs using more sophisticated experimental approaches.

Here we focused on the role of DNA damage (DSBs), particularly in integration of viral DNA. Interestingly, HIV-1 DNA integrated into artificially induced DSBs in an IN-CA–independent manner and DNA damaging agents upregulated the infectivity of IN-CA–defective virus. The positive effects of DSBs on viral integration were resistant to raltegravir (RAL), an IN-CA inhibitor. Moreover, Vpr, an accessory gene product of HIV-1, mimicked DNA damaging agents and increased IN-CA–independent viral transduction into monocyte-derived macrophages (MDMs). Even when the catalytic activity of IN was impaired, infectious secondary virus was generated without any mutations that yielded phenotypes resistant to RAL.

Based on these observations, we propose that the ATM-dependent mode of DSB-specific integration of viral DNA and the Vpr-induced DSBs are novel targets for anti-HIV compounds that inhibit viral transduction into MDMs, a persistent reservoir of HIV-1 infection.

## Results

### HIV-1 integrates into the sites of artificially induced DSBs

**Figure 1:**
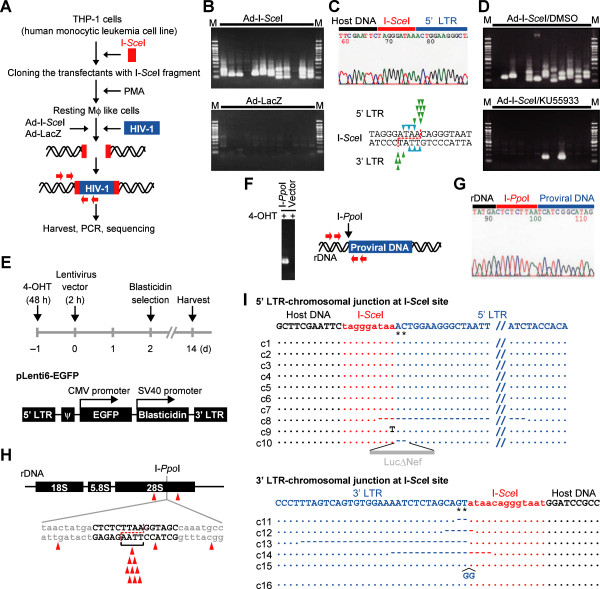
**HIV-1 DNA integrates into a DSB site.** (**A**) An experimental procedures for (**B**)–(**D**) and (**I**). Red arrows indicate the primers used in (**B**) and (**D**). (**B**) PCR amplification of WT provirus DNA integrated in the I-*Sce*I site. Each lane depicts each result of twelve samples independently infected with WT virus and Ad-I-*Sce*I (upper panel) or Ad-LacZ (lower panel). M, molecular marker. (**C**) Upper panel, representative sequencing chromatogram of the PCR amplicon in samples, which were shown in upper panel of (**B**). Lower panel, summary of viral DNA integration sites. The 18-bp recognition sequence of the I-*Sce*I site is shown. When digested with I-*Sce*I, a 3^′^-protruding end of 4 nucleotides is formed (dotted red line). Each arrowhead indicates an actual integration site of viral DNA in samples shown in (**B**). Integration sites were identified on most of clones except for two clones, which are indicated by arrowheads with a horizontal bar. (**D**) Effect of KU55933 on viral DNA integration into the I-*Sce*I site. (**E**) Schematic outline of the I-*Ppo*I-PCR experimental design in (**F**)–(**H**) (Top panel). The lentiviral vector was used in this study (bottom panel). (**F**) PCR amplification of lentiviral vector inserted in the I-*Ppo*I site. Primers are shown by red arrows (**G**) A representative result of sequence analysis of proviral DNA integrated in the I-*Ppo*I site. (**H**) Summary of integration sites of the lentiviral vector. Each arrowhead depicts each result of independent clones. The dotted line indicates I-*Ppo*I site with a 3^′^-protruding end of 4 nucleotides. (**I**) Summary of the I-*Sce*I-PCR sequence data. A representative nucleotide sequence was shown at the top of each panel. Asterisks indicate the pAC that would be normally removed during IN-mediated integration (see Additional file 1: Figure S2). Dots indicate identical sequence to that of the representative sequence. Dashes indicate deleted nucleotides.

To understand the roles of DSBs in integration of viral DNA into macrophages, we established a system using THP-1 cells, a human monocytic leukemia cell line that differentiates into macrophage-like cells after treatment with phorbol myristate acetate (PMA) (Figure 1A) [21]. We transfected THP-1 cells with plasmid DNA that contained the recognition sequence for I-*Sce*I, a rare-cutting endonuclease [22] and obtained clones with the I-*Sce*I site after drug selection. Using the experimental procedures outlined in Figure 1A, the frequency of viral DNA integration into I-*Sce*I sites was evaluated. After PMA-treated cells were infected with VSVG-pseudotyped WT virus (NL-Luc-E(−)R(−)) together with adenovirus-expressing I-*Sce*I, provirus DNA was detected in the I-*Sce*I provirus (Ad-I-*Sce*I) site or its vicinity (Figure 1B, Additional file 1: Figure S1A). PCR amplification targeting the junction of the I-*Sce*I site and the 5^′^-end of the integrated proviral DNA (Figure 1A) selectively generated PCR amplicons from the Ad-I-*Sce*I-infected samples (compare the upper and lower panels of Figure 1B). Sequence analysis of several independent clones detected the presence of provirus DNA in the I-*Sce*I site (Figure 1C, each arrowhead indicates the integration site of individual clones analyzed). Notably, KU55933 blocked I-*Sce*I site-targeted integration (Figure 1D, lower panel) [14].

Similar results were obtained using a different system with another rare-cutting endonuclease, I-*Ppo*I (Figures 1E–H and Additional file 1: Figure S1B). The recognition sites of I-*Ppo*I are present in the human genome, although the mammalian genome has no gene that encodes the enzyme [23]. In this experiment, we used a lentiviral vector to ensure the generality of our observations (Figure 1E). As shown in Figure 1F, the viral DNA reproducibly integrated into the I-*Ppo*I site, which was confirmed by PCR amplification and sequence analysis (Figure 1G and H). The data clearly indicated that the viral DNA was inserted in the DSB sites.

### Integration into DSB sites was independent of the catalytic activity of integrase

**Figure 2:**
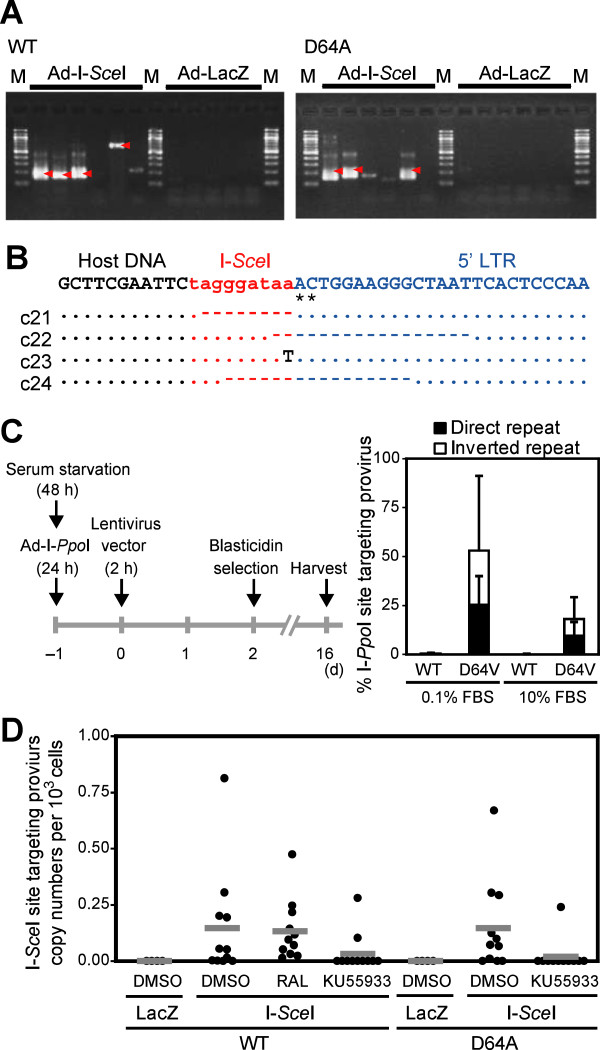
**Frequent integration of the IN-CA defective virus into the DSB site.** (**A**) PCR amplification of provirus DNA integrated into the I-*Sce*I site after infection of WT virus (left) or NL-Luc-IN-D64A-E(−)R(−) virus (D64A virus) (right). PMA-treated THP-1/I-*Sce*I cells were used. Each lane shows an independent result that was obtained from cells cultured in a single well of 6-multiwell. For each test group, six wells were independently infected with viruses. M, molecular marker. Arrowheads indicate amplicons of viral DNA integrated in the I-*Sce*I site, which was further confirmed by sequence analysis. (**B**) Sequence data of D64A provirus DNA that was integrated in the I-*Sce*I site. A representative result is shown at the top. Asterisks indicate the pAC. Dots indicate identical nucleotides to those of the representative sequence. Dashes indicate deleted nucleotides. (**C**) Experimental protocol for evaluating the frequency of viral integration into the DSB site. I-*Ppo*I-qPCR and EGFP-qPCR analyses were done for quantification of I-*Ppo*I site-specific and total proviral DNA copy numbers, respectively. Representative data of two independent experiments was shown. Error bars, s.d. of triplicate assays. (**D**) Evaluation of I-*Sce*I site-targeting efficiency. PMA-treated THP-1/I-*Sce*I cells were infected with WT or D64A virus for 2 h, and cells were harvested 48 hpi for the I-*Sce*I-qPCR analysis (see Methods section). To cleave the I-*Sce*I site, cells were infected with the Ad-I-*Sce*I at an MOI of 100 from 1 h post HIV-1 infection. Treatment with RAL and KU55933 was conducted from −2 h to 48 hpi. Effects of RAL and KU55933 were evaluated on 11 samples that were prepared from three independent experiments. Each dot indicates copy numbers of provirus DNA that had integrated in the I-*Sce*I site in 10^3^ cells, which were infected as a single test sample.

Interestingly, analysis of the nucleotide sequence of the viral DNA inserted in the I-*Sce*I site revealed that both the 5^′^- and 3^′^-long terminal repeat (LTR) ends of the provirus DNA had adenine and cytidine (pAC) dinucleotides (Figure 1I) [6], suggesting that the viral DNA integrated into DSBs in an IN-CA–independent manner (Additional file 1: Figure S2). To confirm this, similar experiments were performed using D64A mutant virus, which is defective in integrase, co-infected with Ad-I-*Sce*I (Figure 2A). PCR amplification followed by sequence analysis consistently detected the presence of pAC in the 5^′^ ends of the integrated viral LTR (Figure 2B).

We then estimated the frequency of viral integration into the DSB sites in the total number of provirus DNA. Intriguingly, we observed that more than half of the integrated D64V lentiviruses were present in the I-*Ppo*I site (approximately 53%) when viral infection was conducted using HT1080 cells that had been cultured in 0.1% FBS (Figure 2C, Additional file 1: Figure S3A). In contrast, the DSB-specific integration of the viral DNA was reduced to approximately 18% in a similar experiment performed in the presence of 10% FBS. FACS analysis of HT1080 cells that had been pulse-labeled with BrdU revealed that the population of cycling cells decreased from 43% to 18% when cells were cultured in 10% and 0.1% FBS, respectively (Additional file 1, Figure S3B). The data indicated that the cellular conditions had a large influence on the rate of viral integration into DSB sites. Of note, no remarkable integration of WT virus into the DSB site was detected under any conditions of cell culture with different concentrations of FBS (Figure 2C). These data suggested that the IN-CA–defective virus was the main target of capture by the DSB sites.

To accurately determine the exact rate of DSB-specific integration of viral DNA, we developed a system for quantitative I-*Sce*I-PCR (I-*Sce*I-qPCR) analysis of the provirus DNA and investigated whether viral DNA integration into the I-*Sce*I site was influenced by RAL (see experimental procedures in Methods section). As shown in Figure 2D, RAL did not attenuate the DSB-specific integration of WT viruses in PMA-treated THP-1 cells (a single dot indicates each result of 11 samples in three independent experimental groups). In contrast, KU55933 efficiently blocked the DSB-specific integration of WT and D64A viruses (Figure 2D). These data suggest that capture of viral DNA in the DSB sites was selectively induced in an IN-CA–independent manner, which was ATM-dependent.

### DNA damaging agents upregulate IN-CA–independent viral integration

**Figure 3:**
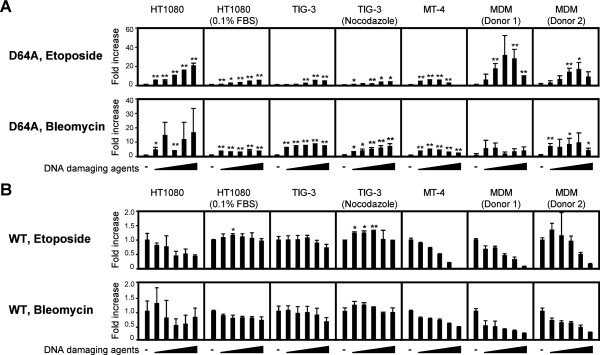
**DNA damage enhances the IN-CA independent infectivity of HIV-1.** Cells were infected with D64A (**A**) or WT (**B**) viruses in the presence of etoposide or bleomycin from 0–48 h post-infection (hpi). After 48 h, cell extracts were prepared and subjected to the luciferase assay. The fold increase of the activity after each viral infection with or without DNA damaging agents was shown. In experiments using cell lines, representative data from one of repeated experiments was shown. Results are presented as mean ± s.d. of triplicate assays. All cells except for MT-4 cells were treated with 0, 0.625, 1.25, 2.5, 5, 10 μM etoposide or 0, 1.25, 2.5, 5, 10, 20 μM bleomycin. MT-4 cells were treated with 0, 0.039, 0.078, 0.156, 0.313, 0.625 μM etoposide or 0, 0.078, 0.156, 0.313, 0.625, 1.25 μM bleomycin. Raw data of luciferase activity was shown in Additional file 1: Figure S4. *, *P* < 0.05; **, *P* < 0.01.

Next, we examined the effects of the DNA damaging agents etoposide and bleomycin on viral infection. As shown in Figure 3A, both compounds increased the infectivity of D64A virus in all cells examined, which included MDMs and various human cell lines. However, the positive effects of these compounds were not consistently observed in WT virus, although they ectopically enhanced the frequency of viral transduction (Figure 3B, Additional file 1, Figure S4), i.e., etoposide enhanced the infectivity of WT virus in serum-starved HT1080 cells and nocodazole-treated human primary fibroblasts (TIG-3) (Figure 3B, upper panels, Additional file 1, Figure S4) [24]. However, it had no positive effects when cells were cultured in the presence of 10% FBS (Figure 3B, upper panels, Additional file 1, Figure S4). In addition, bleomycin had no positive effects on the infectivity of WT virus under any culture conditions (Figure 3B, lower panels, Additional file 1, Figure S4). These data indicate that the effects of DNA damage on viral transduction are only observable when combined with the IN-CA–defective virus, or they are obscured by the infectivity of the WT virus.

### DSBs enhanced viral transduction at the integration step of viral infection

**Figure 4:**
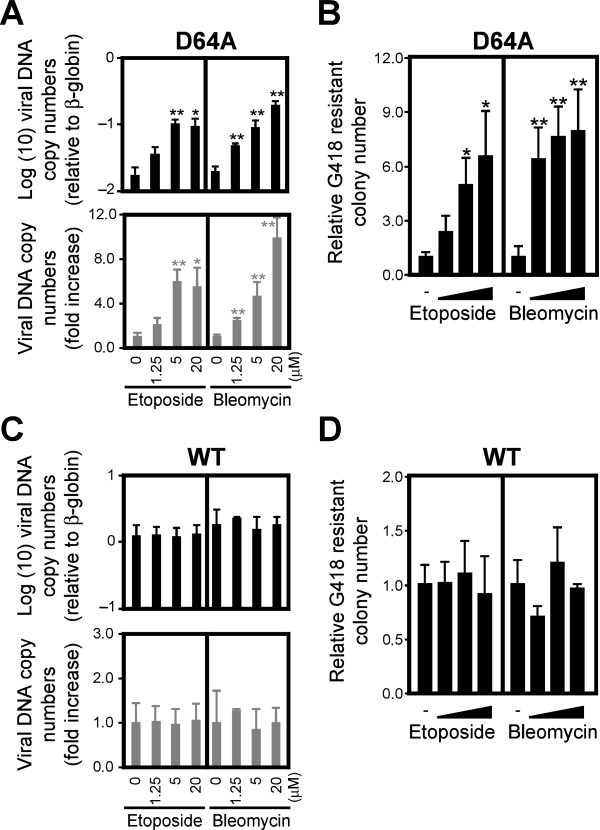
**DNA damage enhances the integration rate of HIV-1.** Serum-starved HT1080 cells were infected with D64A (**A**) or WT (**C**) viruses in the presence of etoposide or bleomycin from 0–24 hpi. After 48 h, genomic DNA was extracted and subjected to qPCR. Relative copy numbers of HIV-1 DNA to β-globin were estimated (top) and the fold increase of HIV-1 DNA copy number compared to control infection that was conducted without DNA damaging agents (bottom) were calculated. For colony formation assay, VSVG-pseudotyped D64A (NL-Neo-IN-D64A-E(−)R(−)) (**B**) or WT (NL-Neo-E(−)R(−)) (**D**) viruses, which had the neomycin resistant gene (Neo^R^), were used. HT1080 cells were treated with various doses of etoposide or bleomycin for 24 h, which were added at the same time of viral infection. After selection with 600 μg/mL of G418, numbers of Neo^R^ colonies were counted. Numbers of Neo^R^ colonies were normalized by plating efficiency. Error bars, s.d. of triplicate assays. *P < 0.05; **P < 0.01.

We quantified the integrated DNA copy numbers to clarify the roles of DSBs in IN-CA–independent viral transduction in greater detail. We used serum-starved HT1080 cells to minimize the possible effects of DSBs generated spontaneously during DNA replication. A quantitative PCR (qPCR)-based assay demonstrated that treatment with 1.25–20 μM etoposide or bleomycin significantly increased the number of integrated viral DNA copies (Figure 4A). We performed a colony formation assay to further demonstrate the effects of DNA damaging agents on viral transduction. As shown in Figure 4B, treatment with DNA damaging agents significantly increased the number of drug-resistant colonies, indicating that DSBs promoted the integration of D64A virus (Figure 4B). In contrast, these compounds had no obvious effects on the integration of WT virus (Figure 4C and D). Although it has been reported that DSBs augment viral replication during multiple steps [15], our observations suggested that they enhance the integration step of viral DNA, which is a pivotal step in viral transduction.

### DSB-dependent viral integration induced minor structural alterations in provirus DNA but generated infectious progeny viruses

It has been proposed that a non-homologous end-joining (NHEJ) pathway is involved in the repair of the gaps formed during viral integration [25] and that the DSB-specific integration of provirus DNA is susceptible to structural alterations [26]. To evaluate this, we quantified the frequency of structural modifications with provirus DNA using linear amplification mediated-PCR (LAM-PCR), followed by nucleotide sequence analysis [27]. When cells were infected with the virus in the presence of RAL, insertions and deletions in the 5^′^-LTR region were detected in 70.6% and 35.3% of cells, respectively (Table 1). In contrast, only 5% of the integrants were positive for structural alterations when infected in the presence of dimethyl sulfoxide (DMSO, solvent control) (Table 1 and Additional file 1: Figure S5). The data implicated that viral integration in the presence of RAL is susceptible to disruption of provirus DNA structures, which abrogated the production of secondary viruses.

**Table 1:** HIV-1 DNA integration sites and host-virus junction sequence

**Seq ID**	**Inhibitor**	**Chr**	**Locus**	**Strand**	**RefSeq gene**	**Insertion**	**Deletion**
100902	DMSO	20	33371451	plus	NCOA6	-	-
100903	DMSO	17	35993714	minus	DDX52	-	-
100904	DMSO	3	156651874	minus	LEKR1	-	-
100906	DMSO	20	60900963	minus	LAMA5	-	-
100907	DMSO	3	19680267	minus	DLG1	-	-
100910	DMSO	4	150767744	plus	-	-	-
100912	DMSO	9	131400460	plus	WDR34	-	-
100914	DMSO	1	156020423	minus	UBQLN4	11bp (ACAGCAGTTAG)	37 bp
100916	DMSO	17	43551920	minus	PLEKHM1	-	-
100919	DMSO	20	2467413	minus	ZNF343	-	-
100921	DMSO	10	114725221	minus	TCF7L2	-	-
106501	DMSO	4	87017147	plus	MAPK10	-	-
106502	DMSO	2	98458616	minus	TMEM131	-	-
106505	DMSO	15	44399565	minus	FRMD10	-	-
106506	DMSO	2	197544261	plus	CCDC150	-	-
106510	DMSO	7	333212296	minus	BBS9	-	-
106513	DMSO	2	230499770	plus	DNER	-	-
106520	DMSO	17	38682989	plus	CR597260	-	-
106521	DMSO	19	17213409	minus	MY09B	-	-
106524	DMSO	6	33219360	plus	VPS52	-	-
100925	RAL	9	33033919	plus	DNAJA1	-	9 bp
100931	RAL	14	44024820	minus	-	-	20 bp
100937	RAL	2	102387409	plus	MAP4K4	9 bp (GACACTTAG0	-
100940	RAL	5	176735496	plus	MXD3	2 bp (AC)	-
100944	RAL	3	105267040	plus	-	21 bp (AATAAAAAGGTACAAATAGAC)	-
106525	RAL	14	87606685	minus	-	6 bp (TCATAA)	3 bp
106530	RAL	22	36006496	plus	MB	1 bp (A)	9 bp
106534	RAL	7	130544996	minus	CR618431	10 bp (TTGTAATTAC)	-
106537	RAL	1	63779999	minus	BC040309	16 bp (AAAGAAAAGGGGGGAC)	-
106538	RAL	3	190624485	minus	-	-	4 bp
106540	RAL	3	85084717	minus	CADM2	-	-
106542	RAL	18	40381428	minus	RIT2	2 bp (CA)	1 bp
106558	RAL	6	52427867	minus	TRAM2	-	-
106574	RAL	9	18460635	plus	-	9 bp (ACACCTAAT)	-
106582	RAL	1	6482403	minus	HES2	4 bp (GGAC)	-
106588	RAL	11	28954901	plus	-	4 bp (GGAC)	-
106596	RAL	7	92394729	plus	CDK6	4 bp (GGAC)	-

HIV-1 DNA integratio sites were determined by LAM-PCR. LAM-PCR sequence were aligned to the human genome reference (GRCh37/hg19, Feb. 2009, UCSC). Chr, chromosome.

**Figure 5:**
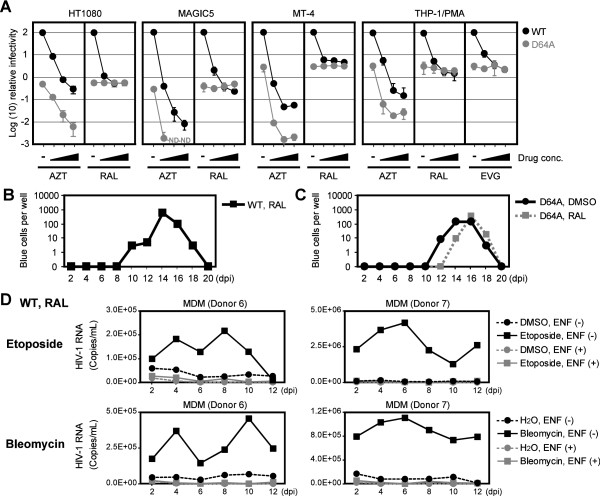
**IN-CA deficient virus can produce infectious progeny virus.** (**A**) Effect of RAL on the infectivity of WT and D64A viruses. After infection with VSVG-pseudotyped WT (NL-Luc-E(−)) or D64A (NL-Luc-IN-D64A-E(−)) virus in the presence of AZT, RAL or EVG, cells were harvested at 48 hpi and subjected to luciferase assay. Relative luciferase activity compared to a control sample, in which WT virus was infected without any compounds, were plotted. Concentration of AZT was 0, 1, 10 and 100 μM, whereas concentrations of RAL and EVG were 0, 0.1, 1 and 10 μM. Black circles, WT virus; gray circles, D64A virus; ND, not detected. Representative data of two independent experiments was shown. Error bars, s.d. of triplicate assays. (**B**) Functional evaluation of progeny viruses generated after IN-CA independent infection. MT-4 cells were infected in the presence of RAL with replication-competent WT virus (NL4-3). Then conditioned medium was harvested every 2 d, and infectivity of progeny virus present in the conditioned medium was evaluated using MAGIC5 cells. (**C**) Similar experiment with (**B**) was done using D64A (NL-IN-D64A) virus. Representative results of three independent experiments are shown. (**D**) Secondary virus generated from MDMs infected with virus in the presence of RAL. MDMs were infected with a replication-competent NL4-3 virus with an *env* gene, which was derived from R5-tropic ADA virus (NL-ADA-R(−)). Then, HIV-1 RNA copy number in the conditioned medium was quantified by RT-qPCR analysis. To evaluate effects of DNA damaging agents, 2.5 μM etoposide or 1.25 μM bleomycin were added from 0–2 dpi. To exclude the possibility that carry-over virions, which were remnant viruses that could not be completely removed after the initial infection, we included control sample, in which a fusion inhibitor enfuvirtide (ENF) was continuously added from 0 dpi to the end of assay.

To clarify this possibility, we investigated the effects of RAL on single-round viral infection using several cell lines. As shown in Figure 5A, we found that the infectivity of the WT virus was significantly attenuated by RAL, i.e., viral infection was reduced to 0.2% and 3.8% when 10 μM RAL was used to treat MAGIC5 cells and MT-4 cells, respectively. However, these values were the same with D64A virus, which suggests that restricting IN-CA could not block viral infection completely. This suggestion was supported by tests using azidothymidine (AZT, an RT inhibitor), which further blocked the infectivity of D64A virus. Importantly, the same results were obtained using elvitegravir (EVG, another IN-strand transfer inhibitor) in PMA-treated THP-1 cells (Figure 5A).

These observations strongly suggest that the WT virus can replicate in the presence of RAL, although the potential for viral replication is low and at similar level to IN-CA–defective virus. To test this possibility, we infected MT-4 cells with a replication-competent virus in the presence of RAL and examined the production of the progeny virus using MAGIC5 cells (HeLa/CD4, CCR5, LTR-β-gal) [28]. As shown in Figure 5B, we observed viral replication with the WT virus, although RAL was continuously added in the culture medium (one representative result from three independent experiments is shown with other experimental data in Additional file 1: Figure S6A). To exclude the possibility that the secondary virus possessed mutations that could overcome the inhibitory effects of RAL, we tested the viral RNA recovered from the culture supernatants. Analysis of the nucleotide sequences of 10 progeny viruses revealed that all clones had no reported mutations related to RAL-resistant phenotypes (Table 2, Additional file 1, Table S1 and Figure S7) [29-32]. A similar experiment was performed using D64A virus. Again, we observed reproducible viral replication in the presence or absence of RAL (Figure 5C, Additional file 1: Figure S6B). Analysis of the nucleotide sequence of the progeny virus RNA revealed that a single clone of the 10 viruses analyzed was positive for a reported mutation linked to a RAL-resistant phenotype (M154I in Table 2, Additional file 1, Table S1). However, the other nine clones were free of such mutations. In addition, no WT virus revertants were detected. It is interesting to note that MT-4, a cell line infected with human T cell leukemia virus, expresses Tax, a viral protein. One possible explanation for the efficient IN-CA independent viral infection is due to DNA damage that is induced by the biological activity of Tax [33,34].

**Table 2:** **IN mutations of NL-ADA or NL-IN-D64A-ADA viruses in Figure**5**B and**5**C**

**Virus**	**Clone #**	**RNA**	**Amino acid**
WT/RAL	#1	-	-
	#5	-	-
	#6	-	-
	#7	-	-
	#8	-	-
	#9	-	-
	#10	-	-
	#2	A144G	E48E (Silent)
	#3	A87G	P29P (Silent)
	#4	A731G	R244K
D64A/DMSO	#1	A191C	D64A
	#2	A191C	D64A
	#3	A191C	D64A
	#4	A191C	D64A
	#7	A191C	D64A
	#9	A191C	D64A
	#5	A191C, A772G	D64A, K258E
	#6	A191C, U842A	D64A, V281E
	#8	A38G, A191C, G462A	E13G, D64A, M154I
	#10	A191C, A279, A809G	D64A, T93T (Silent), D270G
D64A/RAL	#1	A191C	D64A
	#4	A191C	D64A
	#8	A191C	D64A
	#10	A191C	D64A
	#2	A191C, G806C	D64A, R269T
	#3	A191C, A718G	D64A, K240E
	#5	A191C, G829U	D64A, G277C
	#6	A191C, U598C	D54A, I200T
	#9	A191C, G462A	D64A, M154I
	#7	G7A, G39A, G103A, A191C	D3N, E13E (Silent), E35K, D64A

Nucleotide sequence of progeny viral RNA was analyzed. In two of 20 clones examined, we identified M1541, which was reported as a mutation related to RAL-resistant phenotype [30].

After establishing that RAL-resistant viral replication could be induced in MT-4 cells, we investigated whether the same mode of viral infection can occur in MDMs. We detected no apparent replication of infectious secondary virus in MDMs, which were infected in the presence of RAL. However, viral replication was detected when DNA damaging agents were treated at the same time as the viral infection (Figure 5D). Importantly, the addition of enfuvirtide (ENF), a fusion inhibitor, completely abolished the detection of the viral RNA, which indicated that the detected virus was not a remnant of the initially infected virus and that it was a progeny virus. Similar results were obtained in independent experiments using MDMs prepared from a different donor. These data and the absence of reported mutations in these viral RNA showed that DSBs promoted productive viral transduction even in the presence of RAL.

**Figure 6:**
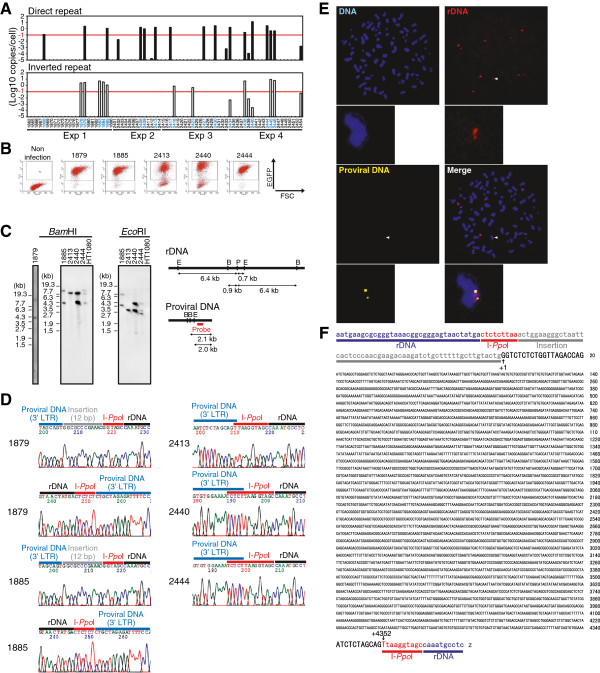
**Detection of intact provirus DNA in the DSB site.** (**A**) I-*Ppo*I-qPCR screening of cell clones containing provirus DNA in I-*Ppo*I site. HT1080 cells were infected with Ad-I-*Ppo*I at an MOI of 30 in the medium with 0.1% FBS. After 24 h, cells were further infected with lentiviruses (VSVG-pseudotyped Lenti6-EGFP-D64V) also under serum-starved conditions. Two h later, medium was changed with fresh one with 0.1% FBS. On the next day, medium was replaced with a complete medium with 10% FBS. Blasticidin-resistant colonies were isolated and I-*Ppo*I site targeting provirus was detected by I-*Ppo*I-qPCR. The threshold of detecting provirus integrated as direct or inverted repeat orientation was −1 log(10) copies/cell (indicated in red horizontal lines). (**B**) EGFP expression analysis. Cells containing the proviral DNA in I-*Ppo*I site in (**A**) were further analyzed for the expression of EGFP by flow cytometer. (**C**) The estimation of proviral DNA copy number. Copy numbers of provirus DNA in EGFP-positive clones, shown in (**B**), were analyzed by Southern blot by using a part of DNA fragment of the lentiviral vector as a probe. Genomic DNA extracted from each clone was digested with *Bam*HI or *Eco*RI prior to electrophoresis. Restriction maps are shown (right panel). B, *Bam*HI; E, *Eco*RI; P, I-*Ppo*I. Of note, clone #2413 possessed a single copy of provirus DNA. (**D**) Sequence analysis of lentiviral vector integrated in the I-*Ppo*I site. EGFP-positive clones shown in (**B**) were subjected to sequence analysis. I-*Ppo*I-PCR amplicons were directly used as a template for sequence analysis. (**E**) FISH analysis of the #2413 clone. (**F**) Nucleotide sequence of intact proviral DNA present in the DSB site. The proviral DNA of #2413 clone was sequenced and whole nucleotide sequence data was shown. In #2413 clone, no structural alternations of provirus DNA were detected.

Based on these experiments, we expected that DSB site may capture and incorporate virus DNA as a structurally intact form. To obtain direct evidence for this possibility, we analyzed the nucleotide sequences of the provirus DNA integrated in the DSB site. In these experiments, serum-starved HT1080 cells were co-infected with an Ad-I-*Ppo*I and an IN-defective lentiviral vector (Lenti6-EGFP-D64V), which contained a blasticidin-resistant gene. After infection, the blasticidin-resistant cells were selected and cloned, and the lentivirus-infected cell clones were screened using I-*Ppo*I-qPCR. We isolated a total of 74 clones and obtained 10 (13.5%), five (6.8%), and five (6.8%) clones, which contained proviral DNA at the I-*Ppo*I site in direct, inverted, or both direct and inverted orientations, respectively (Figure 6A). Of these, five clones were EGFP-positive (Figure 6B) and the proviral DNA was integrated only into the I-*Ppo*I site in one of these clones (Figure 6C, D, clone #2413). This was further confirmed by fluorescent *in situ* hybridization (FISH) analysis, which detected provirus DNA in a single locus in the genome (Figure 6E). Sequence analysis of the provirus DNA of clone #2413 finally identified an intact viral DNA structure with the flanking nucleotide sequence of the I-*Ppo*I site (Figure 6F). The data indicated clearly that the structurally intact viral DNA could integrate into the DSB site.

### Vpr mimicked DSBs and enhanced the IN-CA–independent viral transduction into resting macrophages

**Figure 7:**
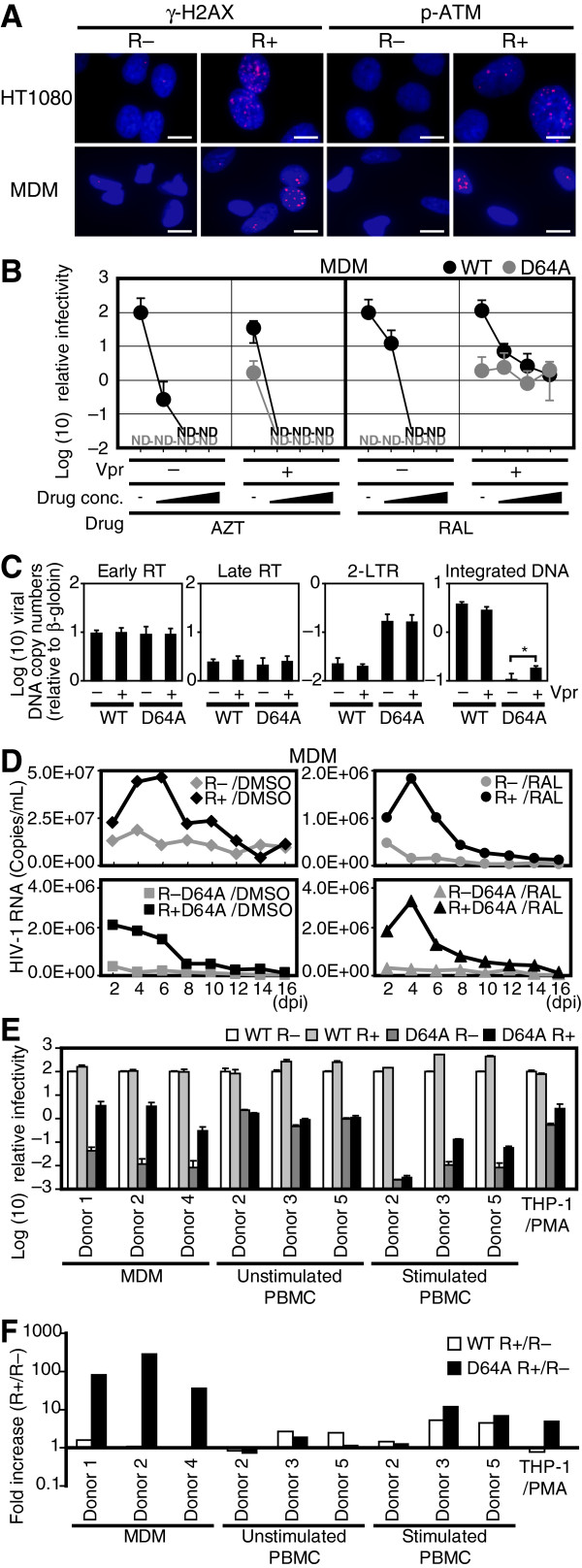
**Vpr mimics DNA damaging agents, and enhances the IN-CA independent macrophage infection.** (**A**) Vpr induces DNA damage cellular signals in MDMs. HT1080 cells or MDMs were infected with VSVG-pseudotyped R– virus (NL-Luc-E(−)R(−)) or R+ virus (NL-Luc-E(−)), and then analyzed immunochemically. Bars = 10 μm. (**B**) Effect of RAL on the infectivity of WT and D64A viruses. MDMs were infected with WT or D64A viruses in the presence of AZT or RAL. The cells were harvested at 48 hpi and subjected to luciferase assay. Relative luciferase activity values to WT R– infectivity are shown. Black circles, WT; gray circles, D64A; ND, not detected. Error bars, s.d. of triplicate assays. (**C**) Effects of Vpr on the integration of viral DNA into the host genome. Serum-starved HT1080 cells were infected with VSVG-pseudotyped IN WT or D64A mutant virus with or without Vpr. After 48 h, infected cells were subjected to qPCR analysis. Error bars, s.d. of triplicate assays. *P < 0.05. (**D**) HIV-1 replicates in MDMs in the presence of RAL. Replication-competent NL4-3 with an intact *env* gene derived from R5-tropic ADA viruses (NL-ADA, NL-ADA-R(−), NL-ADA-IN-D64A, and NL-ADA-IN-D64A-R(−)) were infected. Then, copy numbers of HIV-1 RNA in the conditioned medium was quantified by RT-qPCR. **E** and **F**) Positive effects of Vpr on infection of D64A virus into MDMs. Primary cells and cell lines were infected with IN WT or D64A mutant virus with or without Vpr. Cells were harvested at 48 hpi and subjected to luciferase assay. (**E**) Relative luciferase activity values to WT R– infectivity are shown. White bars, WT/R–; light gray bars, WT/R+; dark gray bars, D64A/R–; black bars, D64A/R+. Error bars, s.d. of triplicate assays. (**F**) Fold increase of R+ virus infectivity to R– virus. White bars, WT; black bars, D64A.

*Vpr*, an accessory gene of HIV-1, encodes a 96-amino acid virion-associated nuclear protein with pleiotropic activities, including a cell cycle abnormality during the G2/M phase, enhanced promoter activity and apoptosis. It has also been proposed that Vpr is important for macrophage infection through the nuclear trafficking of a preintegration complex [35]. Previously, it has been reported that Vpr elicits cellular signals triggered by DNA damage [36-40], which suggests that Vpr promotes IN-CA–independent viral transduction. To test this hypothesis, we checked whether infection with R+ virus induced the DNA damage response in MDMs (Figure 7A). In agreement with our previous observations, infection with R+ virus evoked the cellular response triggered by DNA damage [36,37,41,42]. We investigated the infectivity of R+ virus and observed that Vpr enhanced viral transduction in the presence of RAL, which was blocked by AZT (Figure 7B). Similar to the effect of DSBs, Vpr enhanced the viral infectivity during the integration step (Figure 7C). Moreover, Vpr enhanced the infection of MDMs by D64A virus (Figure 7D and Additional file 1: Figure S8).

To further elucidate the effects of Vpr on the infection of MDMs, we compared the efficiency of viral transduction into MDMs, peripheral blood mononuclear cells (PBMCs), and human cell lines by calculating the fold-increase in the luciferase activity, which reflected the infectivity of each virus (Figure 7E, Additional file 1, Figure S9). As summarized in Figure 7F, the positive effects of Vpr were the most striking when MDMs were infected with D64A virus (D64A/R+ virus). The infectivity of D64A/R+ virus in MDMs was 37.0–265.1-fold higher than that of D64A/R− virus. In contrast, these positive effects were not detected with the WT/R+ virus. Moreover, the positive effects of Vpr were less conspicuous in PBMCs, consistent with previous observations that Vpr functions as a positive factor during viral transduction into MDMs [43]. Combined with previous reports that Vpr activates ATM [36,37] and ATR [38], our observations suggest that the enhanced infectivity of D64A/R+ virus in MDMs is attributable to Vpr-induced DSBs [36,37].

## Discussion

Since it was first postulated that the cellular proteins responsible for DNA damage repair are positively involved in HIV-1 infection [9], roles of DSBs and DNA damage repair enzymes in viral infection have remained controversial [10-14,16-19,44]. However, several lines of evidence have suggested that DSBs have at least two roles in viral infectivity, i.e., direct upregulation of the rate of viral DNA integration into the host genome and the activation of DNA damage repair enzymes, which contribute to multiple steps in HIV-1 infection including repair of the gaps formed during the integration of viral DNA into the host genome [15]. Here we focused on the first possibility and provided experimental data, which showed that DNA damage increased the frequency of viral integration into the host genome.

In particular, we found that DSBs promoted the transduction of D64A virus, which was defective with respect to the catalytic activity of integrase (IN-CA). Moreover, DSBs upregulated the infectivity of WT virus by overcoming the inhibitory effects of RAL, an IN-CA inhibitor. Furthermore, infectious secondary viruses were generated from the provirus DNA formed through IN-CA–independent viral transduction. Our observations were highly consistent with previous reports that the IN-CA–defective virus can integrate into the host genome [45-47]. Ebina *et al.* reported that the integration rate of the IN-CA–defective virus was enhanced by DNA damaging agents such as x-ray irradiation or hydrogen peroxide [48], whereas we showed that DSBs upregulated IN-CA–independent viral integration and promoted the production of secondary viruses, which were competent for subsequent viral infection. Importantly, analysis of the nucleotide sequences of the viral RNA from the secondary viruses showed that there were no revertants to WT virus. Most of the viruses analyzed also had no reported mutations linked to RAL-resistant phenotypes [29-32]. Taken together with observation that RAL could reduce the infectivity of WT virus at a similar level to D64A virus, our data also suggest that currently available IN inhibitors cannot completely block productive viral infection, which is possibly enhanced by DSBs.

The mechanism of DSB-induced upregulation of viral transduction remains elusive but our data suggest that DSB sites provide a platform where viral DNA integrates in an IN-CA–independent manner. When cells were co-infected with HIV-1 virus and an adenovirus that expressed rare-cutting endonucleases such as I-*Sce*I or I-*Ppo*I, we reproducibly observed that the viral DNA was integrated into the corresponding DSB sites. However, interestingly, DSB-site specific viral integration was influenced by viral and cellular factors. First, we observed that targeting of viral DNA to the DSB site was observed mainly during IN-CA–independent viral transduction, although its frequency was low compared with WT virus. Second, it was influenced by the cellular conditions of the target cells, i.e., the frequency of IN-CA-independent viral transduction into DSB sites decreased from approximately 53% to 18% when the concentration of FBS was changed from 0.1% to 10% (Figure 2C). These results and the FACS analysis suggest that this difference may be because the spontaneous DSBs generated during DNA replication also captured viral DNA, which resulted in a decrease in the relative rate of viral integration into artificially induced DSBs.

Interestingly, the DSB-specific integration of DNA fragments has been reported for hepatitis B virus DNA, an adeno-associated viral vector (AAV) [49,50], and Ty1 [51], a DNA retrotransposon of *Saccharomyces cerevisiae.* These observations suggest that the DSB site-specific integration of exogenous DNA fragments is not lentivirus-specific, which also indicates that DSB site-specific integration is dependent on the cellular response to DNA damage. We observed that KU55933, a specific ATM inhibitor, consistently blocked DSB-specific viral integration (Figures 1D and 2D). Interestingly, x-ray irradiation triggers the retrotransposition of long interspersed element 1 in human cells, which is also dependent on ATM [52], implying that a conserved cellular response to DNA damage is functionally involved in the capture of viral DNA in the DSB site.

We detected minor nucleotide deletions of approximately <9 bp in five of six clones of the provirus DNA, which were derived from cells infected with virus in the presence of RAL (Table 1). Such structural alternations would be due to the NHEJ repair system that is involved in viral integration in the presence of RAL. Because it has been reported that provirus DNA with 10-bp deletions from nucleotides +3 to +12 in the 5^′^-LTR remained functional [53], such provirus DNA is likely to be replication competent, although minor modifications in the 5^′^-LTR may be related to reduced expression of viral mRNA, as reported by Ebina *et al.*[48].

Several researchers have proposed that viral mRNA is expressed from non-integrated viral DNA of the IN-CA-defective virus [54,55], whereas Vpr was shown to promote *Nef* mRNA expression from such an extrachromosomal viral DNA [56]. However, our study clearly indicated that Vpr upregulates integration of IN-CA–defective virus into the host genome. The positive effects of Vpr on viral transduction were more prominent in MDMs than in PBMCs, well consistent with reports that Vpr functions as a positive factor during viral transduction into MDMs. Combined with observations that Vpr activates ATM [36,37] and ATR [38] and that macrophages are resistant to DSBs compared with monocytes [20], our data suggest that the enhancement of IN-CA–independent viral transduction into MDMs may be a pivotal role of Vpr in HIV-1 infection.

In summary, our observations may have major importance in the debate on the involvement of cellular factors in viral integration. It has been postulated that DNA damage sensor molecules are involved in the efficient integration of viral DNA. It has also been claimed that DNA damage sensor proteins have no involvement in DNA damage-dependent viral integration. Here we showed that DSBs are particularly important for IN-CA–independent viral transduction and that the effects of DSBs should be analyzed in carefully designed experimental conditions or else their effects are obscured. Collectively, our data suggest that complete prevention of viral integration will require the development of novel compounds that can protect cells from IN-CA–independent viral integration.

## Conclusion

The ATM-dependent mode of the DSB-specific viral DNA integration and Vpr-induced DSBs may be novel targets for anti-HIV compounds that inhibit viral transduction into MDMs, which are a persistent focus of HIV-1 infection.

## Methods

### Plasmid constructs

The vesicular stomatitis virus glycoprotein (VSVG) expression vector pHIT/G [57], the HIV-1 proviral construct pNL4-3 [58], pNL-ADA [59], and the HIV-1 proviral indicator constructs pNL-Luc-E(−) and pNL-Luc-E(−)R(−) [37] have been described previously. To introduce D64A mutation into IN (adenine of nucleotide 4420 to cytosine) to create pNL-IN-D64A, site-directed mutagenesis (QuikChange; Stratagene) was performed using pNL4-3 as a template. To create pNL-ADA-IN-D64A and pNL-Luc-IN-D64A-E(−) that contained D64A mutants, the *Spe*I-*Pfl*MI fragment (nucleotides 1507–5297) of pNL-IN-D64A was replaced with those of pNL-ADA and pNL-Luc-E(−), respectively. To create the Vpr-deficient construct pNL-ADA-R(−), pNL-ADA-IN-D64A-R(−), and pNL-Luc-IN-D64A-E(−)R(−), the *Pfl*MI-*Sal*I fragment (nucleotides 5297–5785) of pNL-Luc-E(−)R(−) was replaced with those of pNL-ADA, pNL-ADA-IN-D64A, and pNL-Luc-IN-D64A-E(−), respectively.

The neomycin-resistant marker expressing vector pNL-Neo-E(−)R(−) was created by inserting a PCR-amplified neomycin-resistant gene into the *Not*I-*Xho*I site of pNL-Luc-E(−)R(−). To create a neomycin-resistant marker expressing D64A, the mutant pNL-Neo-IN-D64A-E(−)R(−) was created by the *Spe*I-*Pfl*MI fragment (nucleotides 1507–5297) of pNL-IN-D64A and replaced with that of pNL-Neo-E(−)R(−). To create pIRES2-EGFP-I-*Sce*I, a pIRES2-EGFP (Clontech)-based plasmid with an I-*Sce*I recognition site, a synthetic double-stranded oligonucleotide (I-*Sce*I-sense and I-*Sce*I-antisense oligonucleotides; see Additional file 1: Table S2) was inserted into the *Eco*RI and *Bam*HI sites of pIRES2-EGFP.

To make the adenoviral vector Ad-I-*Ppo*I, I-*Ppo*I cDNA was amplified from pBabe-HA-ER-I-*Ppo*I using the Adeno-*Ppo*I-*Dra*I-F and Adeno-*Ppo*I-*Dra*I-R primers (Additional file 1: Table S2) and cloned into the *Swa*I site of the pAxCALNLwtit2 cosmid vector (NIPPON GENE). To generate the EGFP-expressing lentiviral vector (pLenti6-EGFP), *EGFP* cDNA from pENTR1a-EGFP was cloned into pLenti6/V5-DEST (Invitrogen) using LR Clonase (Invitrogen). The IN D64V mutation of the gag/pol-expressing plasmid pLP1 (Invitrogen; pLP1-IN-D64V) was introduced using pLP1 as a template with site-directed mutagenesis (QuikChange; Stratagene).

### Cell culture

THP-1, HT1080, HEK293, and HEK293T cell lines were obtained from the RIKEN Cell Bank. TIG-3 (primary human fibroblast cells) and MT-4 cells were obtained from the Health Science Research Resources Bank (Osaka, Japan). HT1080, HEK293, HEK293T, MAGIC5, and TIG-3 cells were maintained in Dulbecco’s modified Eagle’s medium (DMEM) supplemented with 10% fetal bovine serum (FBS). MT-4 cell was maintained in RPMI 1640 supplemented with 10% FBS. To obtain macrophage-like cells, THP-1 cells, maintained in Iscove’s modified Dulbecco’s medium supplemented with 10% FBS, were treated for 2 d with 5.0 × 10^–8^ M PMA. As described previously [60], PMA-treated THP-1 cells were positive for Mac-1, a specific marker of macrophages. Peripheral blood was derived from healthy donors who worked within the institute and gave informed consent. Experimental procedures were approved by the internal review board. PBMCs and MDMs were prepared and cultured as previously described [61]. MDMs were prepared from healthy volunteers who gave informed consents. The experimental protocol was approved by the internal review board.

### HIV-1 and lentiviral vector preparation

The preparation and titration of replication-competent and VSVG-pseudotyped viruses are described elsewhere [36,37,62,63]. The lentiviral vectors were prepared using pLenti6-EGFP and the ViraPower Lentiviral Packaging Mix (Invitrogen) according to the manufacturer’s protocol. Viral supernatants were centrifuged at 120 × *g* for 5 min, filtered through a 0.2-μm filter, and stored at −80°C. To exchange the medium for DMEM supplemented with 0.1% FBS, the viruses were ultracentrifuged at 86,000 × *g* for 1 h.

### Quantitative PCR of provirus DNA

For the quantification of early RT, late RT, 2-LTR circle, and integrated DNA, qPCR was performed as described elsewhere [64,65]. Briefly, cells were harvested at 48 hpi, and genomic DNA was prepared by QuickGene (FujiFilm). For the quantification of early RT, late RT, and 2-LTR circle products, the primers and probe sets M667/AA55/R-U5, M667/M661/R-U5, and MH535/2-LTR-AS/NL4-3-U3 were used, respectively. TaqMan Universal PCR Master Mix with UNG (Applied Biosystems) and ABI7000 (Applied Biosystems) were used according to the manufacturer’s instructions. For Alu-PCR (quantification of integrated DNA), the primer and probe sets first-Alu-F/first-Alu-R/first-gag-R and second-tag-R/2-LTR-S/probe-2 were used for the first and second rounds of qPCR, respectively. The amplification conditions for the first round of PCR, using AmpliTaq Gold 360 Master Mix (Applied Biosystems), were as follows: 95°C for 10 min, followed by 12 cycles at 95°C for 15 s, 60°C for 30 s, and 72°C for 10 min. The second round of qPCR was conducted using TaqMan Universal PCR Master Mix according to the manufacturer’s instructions. To generate a standard curve for Alu-PCR, HEK293T cells (approximately one million cells) were infected with VSVG-pseudotyped NL-Luc-E(−)R(−) virus (200 ng p24), then harvested at 30 d post-infection (dpi), and genomic DNA was prepared. For the quantification of β-globin DNA copy numbers, the primer set globin-F/globin-R was used with SYBR *Premix ExTaq* (TaKaRa). Sequence information for primers and probes is listed in Additional file 1: Table S2.

### Cleavage of I-*Sce*I and I-*Ppo*I sites

Ad-I-*Sce*I and Ad-LacZ were prepared as described previously [36]. PMA-treated THP-1 cells were infected with Ad-I-*Sce*I or Ad-LacZ at 1 h post–HIV-1 infection for 1 h at a multiplicity of infection (MOI) of 100. In Figure 1E–1H, HT1080 cells were transfected with plasmid DNA that encoded a chmeric protein of estrogen receptor-I-*Ppo*I (ER-I-*Ppo*I), and then 4-hydroxytamoxifen (4-OHT) was added to activate the endonuclease and induce DSB. The pAxCALNLwtit2 cosmid vector harboring I-*Ppo*I cDNA was digested with *Bsp*T104I and transduced into HEK293 cells to produce Ad-I-*Ppo*I. The adenoviral vector encoding Cre recombinase, AxCANCre (TaKaRa), was co-infected with Ad-I-*Ppo*I at an MOI of 30 to remove the floxed stuffer between the CAG promoter and I-*Ppo*I cDNA.

### Quantification of the I-*Sce*I site specific viral integration

PMA-treated THP-1 cells were co-infected with WT virus and Ad-I-*Sce*I or Ad-LacZ, and then extracted genomic DNA was subjected to I-*Sce*I-qPCR analysis. I-*Sce*I site targeting integration rate of HIV-1 DNA was estimated by PCR amplification using primer sets pIRES2eGFP+543F/pNL4-3+9207R and pIRES2eGFP+574F/pNL4-3+98R+9173R for the first and second rounds of qPCR, respectively. The amplification conditions for the first round of PCR, using *ExTaq* polymerase (TaKaRa), were as follows: 30 cycles of 98°C for 10 s, 60°C for 30 s, and 72°C for 30 s. The second round of qPCR was conducted using SYBR *Premix ExTaq* polymerase (TaKaRa) according to the manufacturer’s instructions. For the second round PCR template, 1/25 the volume of the first PCR amplicon was used. To prepare a standard sample for the I-*Sce*I-qPCR, the 5'-LTR DNA fragment of HIV-1 was amplified using the pNL4-3+9074F-*Sce*-RI and pNL4-3+9423R-*Bam*HI and cloned into the *Eco*RI and *Bam*HI sites of pIRES2-EGFP (pIRES2-EGFP-5'-LTR). Then, HT1080 cells were transfected with pIRES2-EGFP-5'-LTR and HT1080/pIRES2-EGFP-5'-LTR cell was obtained. By Southern blot and sequence analyses we confirmed that two copies of the DNA fragment of pIRES2-EGFP-5'-LTR vector were present in HT1080/pIRES2-EGFP-5'-LTR diploid cells. Sequence information for primers and probes is listed in Additional file 1: Table S2.

### Quantification of the I-*Ppo*I site specific viral integration

Serum starved HT1080 cells were co-infected with Ad-I-*Ppo*I and lentiviruses, which were generated by pLenti6-EGFP or pLP1-IN-D64V. To estimate I-*Ppo*I site targeting or total integration of the lentiviral vector, I-*Ppo*I-qPCR or EGFP-qPCR was conducted using the TaqMan Universal PCR Master Mix (Applied Biosystems). For I-*Ppo*I-qPCR in the direct or inverted repeat orientation, the primer sets rDNA+11725R/pLenti6+5237F or rDNA+11645F/pLenti6+5237F were used, respectively; pLenti6-LTR was used as the TaqMan probe. For EGFP-qPCR, the primers EGFP-F/EGFP-R and TaqMan EGFP-probe were used. As a standard sample for estimating copy numbers of viral DNA integrated in the I-*Ppo*I site, genomic DNA of HT1080/Lenti6-EGFP-std cells were was used. We have confirmed by Southern blot and sequence analyses that HT1080/Lenti6-EGFP-std cells harbored two copies of Lenti6-EGFP proviral DNA in both orientations in the I-*Ppo*I site. On the other hand, as a standard sample for total provirus DNA, genomic DNA of HT1080/pIRES2-EGFP-5'-LTR cells, which possessed two copies of *EGFP*, were used. Sequence information for primers and probes is listed in Additional file 1: Table S2.

### PCR and sequence analysis

To amplify the host DNA/5'-LTR junction at the I-*Sce*I site, the primer sets pIRES2eGFP+543F/pNL4-3+9207R and pIRES2eGFP+574F/pNL4-3+98R+9173R were used for the first and second rounds of PCR, respectively. To amplify the host DNA/3'-LTR junction at the I-*Sce*I site, the primer sets pIRES2eGFP+1910R/L-M667 and pIRES2eGFP+887R/LambdaT were used for the first and second rounds of PCR, respectively. The amplification conditions for the host DNA/5'-LTR and host DNA/3'-LTR were as follows: 40 cycles for the first round of PCR or 30 cycles for the second round of PCR at 98°C for 10 s, 60°C for 30 s, and 72°C for 30 s and 35 cycles at 98°C for 10 s, 60°C for 30 s, and 72°C for 60 s for the first round of PCR and 30 s for the second round of PCR, respectively. *ExTaq* polymerase (TaKaRa) was used for the PCR. PCR amplicons were used directly or cloned into pCR2.1-TOPO (Invitrogen) as a template for sequence analysis. To amplify the rDNA/lentiviral vector at the I-*Ppo*I site in the direct repeat orientation, the primer sets rDNA+11784R/pLenti6+5208F and rDNA+11747R/pLenti6+5232F were used for the first and second rounds of PCR, respectively. To amplify the rDNA/lentiviral vector at the I-*Ppo*I site in the inverted repeat orientation, the primer sets rDNA+11589F/pLenti6+5208F and rDNA+11612F/pLenti6+5232F were used for the first and second rounds of PCR, respectively. The amplification conditions for the rDNA/lentiviral vector at the I-*Ppo*I site were as follows: 40 cycles for the first and second rounds of PCR at 98°C for 10 s, 60°C for 30 s, and 72°C for 30 s. *ExTaq* polymerase (TaKaRa) was used for PCR. PCR amplicons were used directly or cloned into pCR2.1-TOPO (Invitrogen) as a template for sequence analysis. To analyze the IN mutations of NL-ADA and NL-IN-D64A-ADA viruses in Figure 5B and 5C, viral RNAs were isolated from conditioned medium (14 dpi; WT/RAL and D64A/DMSO, 16 dpi; D64A/RAL) and amplified by primer set pNL+4207F/pNL+5120R and Titan One-Tube RT-PCR Kit (Roche diagnostics). The amplicons were cloned into pCR2.1-TOPO and sequenced. The primers are listed in Additional file 1: Table S2.

### LAM-PCR

To estimate the rate of insertion and/or deletion, the LAM-PCR method was performed as described previously [27,66]. Briefly, 1.0 × 10^6^ HT1080 cells were infected with VSVG-pseudotyped NL-Neo-E(−)R(−) virus (200 ng p24) in the presence of RAL or DMSO, and G418-resistant cells were harvested at 28 dpi and subjected to LAM-PCR. The sequence information for primers is listed in Additional file 1: Table S2.

### Replication assay

To evaluate the production of functional virion from RAL-treated cells, 1 × 10^5^ MT-4 cells were infected with replication-competent NL4-3 or NL-IN-D64A (20 ng p24). After 2 h of the infection, cells were washed with phosphate buffered saline (PBS) twice and suspended in 1.0 mL of medium. To prepare the culture supernatant, three-quarter of the cultures were harvested every 2 d, and the culture was continued by adding 750 μL of the complete medium into each well. From −1 dpi to harvest, MT-4 cells were treated with 10 μM RAL or DMSO. Conditioned medium (100 μL) was added to 1 × 10^4^ MAGIC5 cells [28], and at 48 hpi, cells were stained by X-gal to estimate the number of transduced cells. To estimate HIV-1 RNA copy numbers, 1 × 10^5^ MDMs were infected with NL-ADA, NL-ADA-R(−), NL-ADA-IN-D64A, or NL-ADA-IN-D64A-R(−) (20 ng p24) for 2 h, then washed with medium four times. Three-quarters of the conditioned medium was harvested and replaced with fresh medium every 2 d. From −1 dpi to harvest, MDMs were treated with 10 μM RAL or DMSO. HIV-1 RNA of conditioned medium was purified and subjected to RT-qPCR using the Lenti-X qRT-PCR Titration Kit (TaKaRa). To evaluate the effect of DNA damaging agents, 2.5 μM etoposide or 1.25 μM bleomycin were added to MDMs from 0–2 dpi. To exclude a possibility that detected HIV RNA merely reflect the RNA from carry-over virion, fusion inhibitor ENF, dissolved in phosphate buffer saline PBS, was added from 0 hpi to harvest as a negative control.

### Colony formation assay

To evaluate the effect of DNA damaging agents on the integration rate of D64A mutant virus, serum-starved HT1080 cells (5 × 10^5^ cells) in DMEM with 0.1% FBS were infected with a neomycin-resistant marker expressing VSVG-pseudotyped NL-Neo-IN-D64A-E(−)R(−) virus (100 ng p24) in the presence of 0 (DMSO), 0.625, 1.25, or 2.5 μM etoposide and 0 (water), 0.313, 0.625, or 1.25 μM bleomycin. Cells were selected with G418 from 2 dpi, then stained with Giemsa at 12 dpi. The G418-resistant colony numbers were normalized by plating efficiency, which represented the cytotoxicity of etoposide and bleomycin. The plating efficiencies after treatment of etoposide and bleomycin at 0, 0.625, 1.25, 2.5 μM were 100, 57.2, 26.0 and 19.5%, and 100, 60.4, 68.8 and 60.4%, respectively.

### Immunohistochemical analysis

Detection of phosphorylated histone H2AX (γH2AX) and phosphorylated ATM (pATM) was done, according to the reported method using antibodies against γH2AX (phosphorylated at Ser 139, Millipore, cat no. 05–636) and pATM (Ser 1981, Millipore, cat. no. 05–740) [37]. Briefly, the cells were washed with PBS and fixed with 4% paraformaldehyde in PBS. The fixed cells were permeabilized with 0.2% Triton X-100 in PBS. After treatment with PBS supplemented with 10% goat serum for 30 min, the cells were incubated with antibodies. After 1 h of incubation at 37°C, secondary antibodies conjugated with Alexa 546 (Molecular Probes) were added for 1 h at 37°C. Nuclei were stained by Hoechst33258.

### Luciferase assay

To evaluate the infectivity of viruses, 1.0 × 10^4^ cells were infected with VSVG-pseudotyped luciferase viruses (2 ng p24) for 48 h, then subjected to luciferase assay by using One-Glo (Promega) and a Veritas Microplate luminometer (TURNER BIOSYSTEMS).

### Flow cytometry

To analyze the status of cell cycle, HT1080 cells were labeled with 5 μM BrdU for 30 min and fixed in ice-cold 70% ethanol. Anti-BrdU-fluorescein (Roche Diagnostics) was used to stain the BrdU-labeled cells, according to the manufacturer’s instructions. BrdU-labeled cells were analyzed by BD FACSCalibur flow cytometer (BD Biosciences). To analyze the percentages of EGFP-positive cells, Flow cytometry analysis was performed using a BD FACSCalibur.

### FISH analysis

Metaphase FISH analysis was performed to estimate the proviral DNA copy number and co-localization of proviral DNA and rDNA using lentiviral vector and rDNA specific probes (Chromosome Science Labo Inc.).

### Statistical analysis

Statistical significance was determined by Student’s *t*-test, and values of *P* < 0.05 were considered statistically significant.

## Competing interests

The authors declare no competing financial interests.

## Authors’ contributions

TK and BS designed and performed the work, analyzed the data and wrote the paper. KT and MT contributed reagents and technical advice for the work, and edited the manuscript. YI conceived of the study and supervised the work and wrote the paper. All authors read and approved the final manuscript.

## Supplementary Material

Additional file 1: Figure S1 Southern blot analysis to verify the cleavage of the I-*Sce*I and I-*Ppo*l site. **Figure S2.** A schematic of the strand transfer of HIV-1 DNA to genomic DNA. **Figure S3.** Evaluation of lentiviral infectivity and cell cycle status of serum starved HT1080 cells by BrdU incorportion. **Figure S4.** Raw data in Figure 3. **Figure S5.** The percentage of insertion and/or deletion (InDel) mutations at the host/viral junction. **Figure S6.** Two additional independent data sets in Figure 5B and 5C. **Figure S7.** RAL in 2d cultured conditioned medium is active. **Figure S8.** Additinoal data sets with another donor in Figure 7D. **Figure S9.** Raw data in Figure 7E. **Table S1.** RAL and EVG resistant mutations reported in literature. **Table S2.** List of primers and probes used in this study. **Supplementary methods.** Southern blot analysis. Click here for file
