# Supplementary material for: DNA damage enhances integration of HIV-1 into macrophages by overcoming integrase inhibition
Source: Retrovirology. 2013 Feb 21;10:21. doi: 10.1186/1742-4690-10-21 (PMC3605128; doi:10.1186/1742-4690-10-21)
Supplement: Additional file 1: Figure S1 — Southern blot analysis to verify the cleavage of the I-SceI and I-Ppol site. Figure S2. A schematic of the strand transfer of HIV-1 DNA to genomic DNA. Figure S3. Evaluation of lentiviral infectivity and cell cycle status of serum starved HT1080 cells by BrdU incorportion. Figure S4. Raw data in Figure 3. Figure S5. The percentage of insertion and/or deletion (InDel) mutations at the host/viral junction. Figure S6. Two additional independent data sets in Figure 5B and 5C. Figure S7. RAL in 2d cultured conditioned medium is active. Figure S8. Additinoal data sets with another donor in Figure 7D. Figure S9. Raw data in Figure 7E. Table S1. RAL and EVG resistant mutations reported in literature. Table S2. List of primers and probes used in this study. Supplementary methods. Southern blot analysis. [file 1742-4690-10-21-S1.pdf]

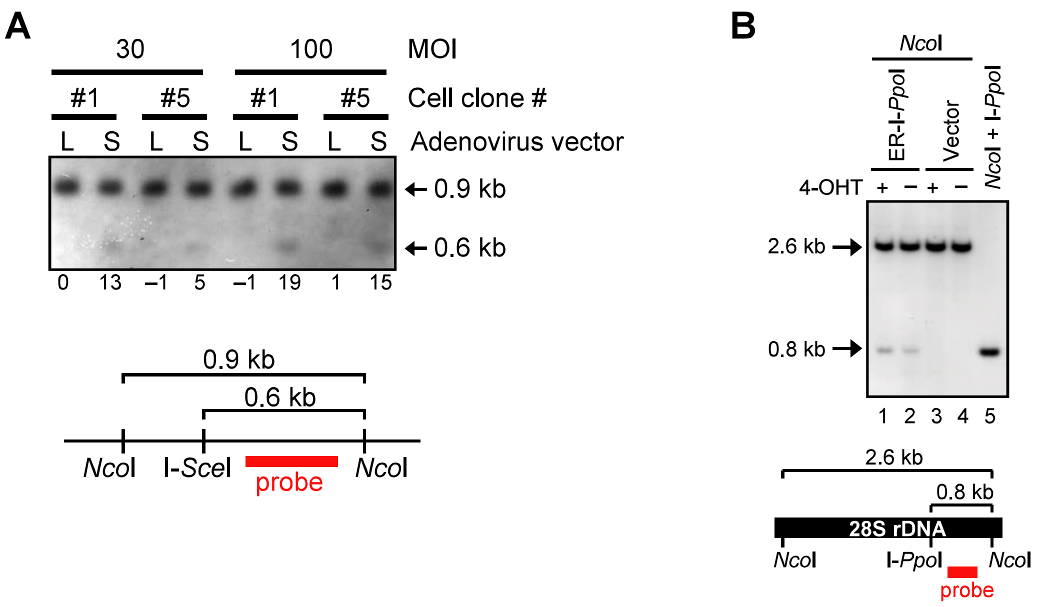

Additional File 1: Figure S1. Southern blot analysis to verify the cleavage of the I-*Sce*I and I-*Ppo*I site. (**A**) Southern blot analysis to verify the cleavage of the I-*Sce*I site. Two independent THP-1/I-*Sce*I cell clones were used, in which the exogenous I-*Sce*I recognition DNA fragment was inserted on chromosomes 14 (clone #1) and 17 (clone #5). I-*Sce*I cleavage generates a 0.6 kb DNA fragment. I-*Sce*I cleavage efficiency was estimated by ImageJ and shown at the bottom of picture. The restriction enzyme map around the I-*Sce*I site is shown (bottom panel). S, Ad-I-*Sce*I; L, Ad-LacZ. (**B**) Southern blot analysis to verify the cleavage of the rDNA I-*Ppo*I site. 4-OHT was added to clonal HEK293T/ER-I-*Ppo*I (Lane 1) or HEK293T/Vector (Lane 3) cell cultures at a final concentration of 1 μM for 24 h to induce the nuclear localization of ER-I-*Ppo*I. Genomic DNA was isolated and digested with *Nco*I prior to agarose gel electrophoresis. I-*Ppo*I cleavage generates a 0.8 kb DNA fragment. Genomic DNA of HT1080 cells digested with *Nco*I and I-*Ppo*I in vitro was used as a positive control (Lane 5). The restriction enzyme map around the 28S rDNA I-*Ppo*I site is shown (bottom panel).

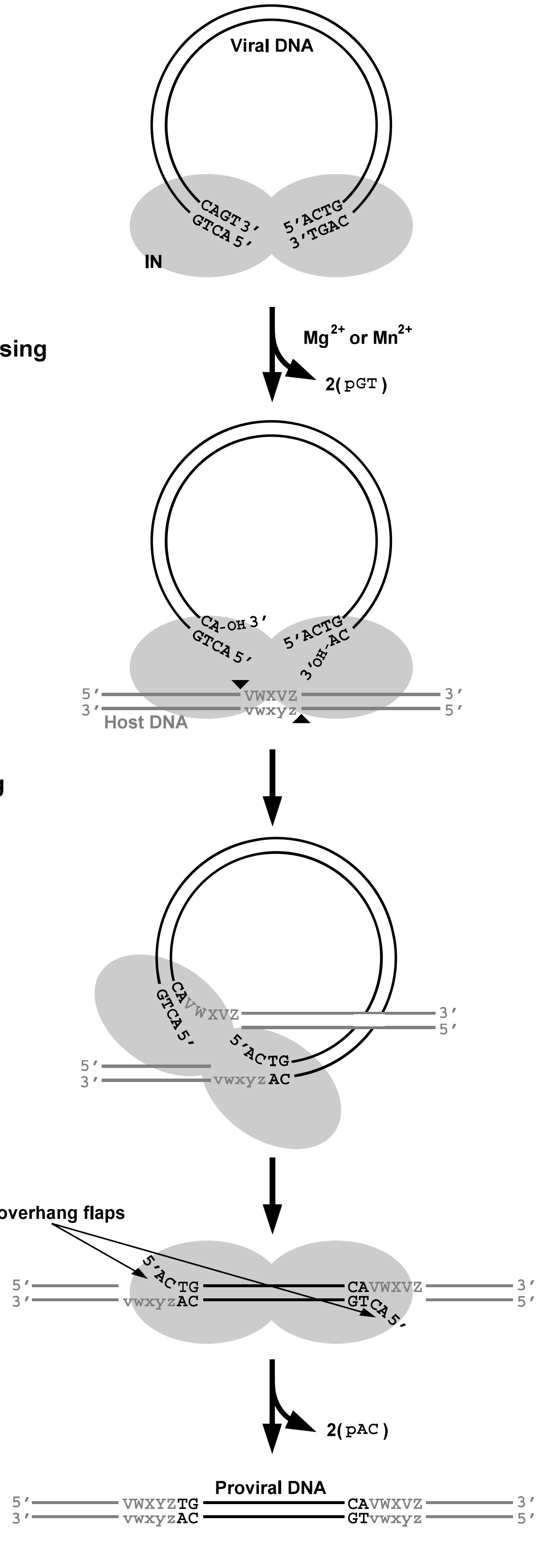

Additional File 1: Figure S2. A schematic of the strand transfer of HIV-1 DNA to genomic DNA. Note that 5' over-hanging flaps are generated at the "Joining" step and are removed during the post-integration "Repair" step, and only the pTG of pACTG in HIV-1 RNA is retained in the proviral DNA inserted into the host genome.

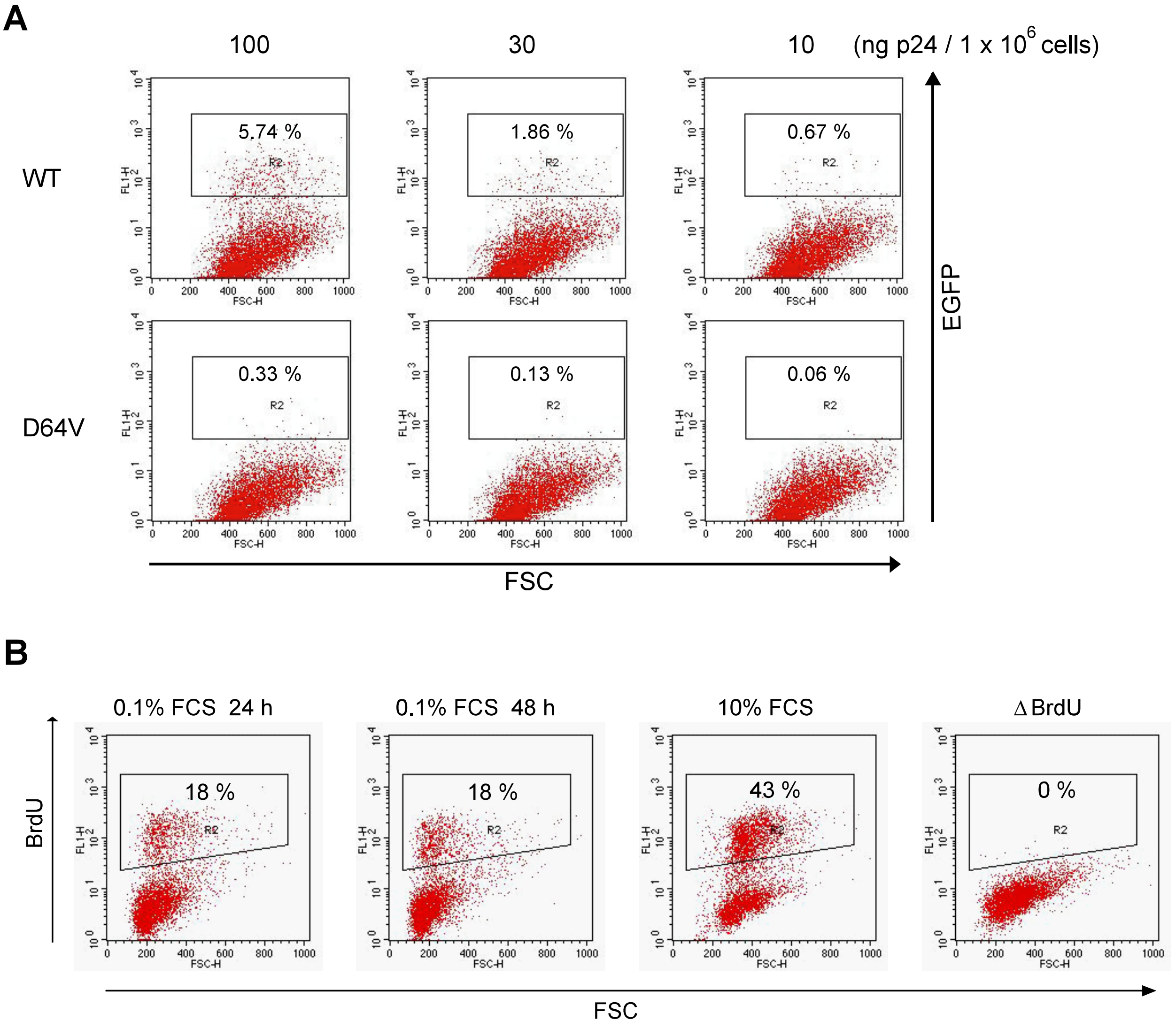

Additional File 1: Figure S3. Evaluation of lentiviral infectivity and cell cycle status of serum starved HT1080 cells by BrdU incorporation. (**A**) HT1080 cells were infected with WT (Lenti6-EGFP-WT) or D64V (Lenti6-EGFP-D64V) lentivirus. The percentages of EGFP-positive cells at 48 h post infection is measured. (**B**) HT1080 cells were serum starved in DMEM supplemented with 0.1% FCS for the indicated time. Cells were labeled with 5 μM BrdU for 30 min. The percentage of BrdU-labeled cells is indicated in each panel. ΔBrdU indicates the non-labeled cells used as a negative control.

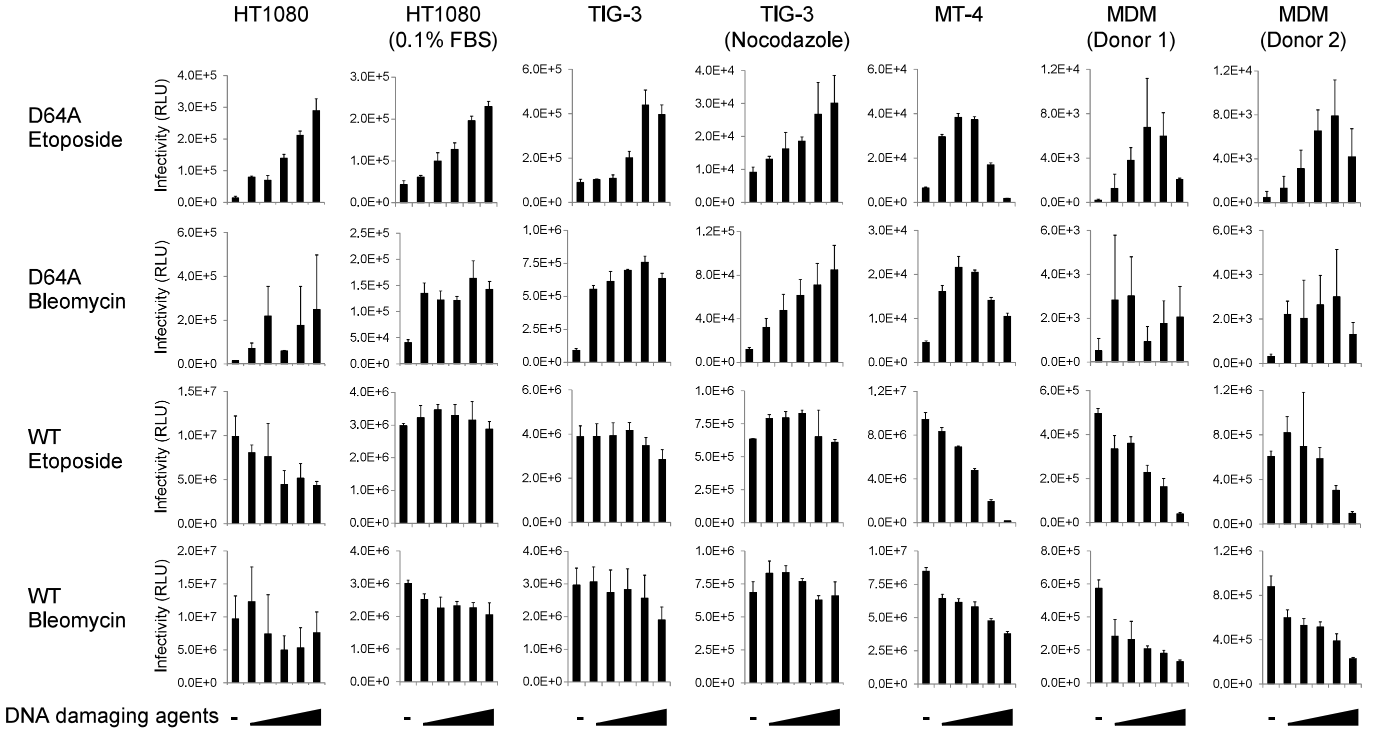

Additional File 1: Figure S4. Raw data in Figure 3. Cells were infected with D64A or WT viruses in the presence of etoposide or bleomycin from 0–48 h post-infection. After 48 h, cell extracts were prepared and subjected to the luciferase assay. In experiments using cell lines, representative data from one of repeated experiments was shown. Results are presented as mean ± s.d. of triplicate assays. All cells except for MT-4 cells were treated with 0, 0.625, 1.25, 2.5, 5, 10 μM etoposide or 0, 1.25, 2.5, 5, 10, 20 μM bleomycin. MT-4 cells were treated with 0, 0.039, 0.078, 0.156, 0.313, 0.625 μM etoposide or 0, 0.078, 0.156, 0.313, 0.625, 1.25 μM bleomycin.

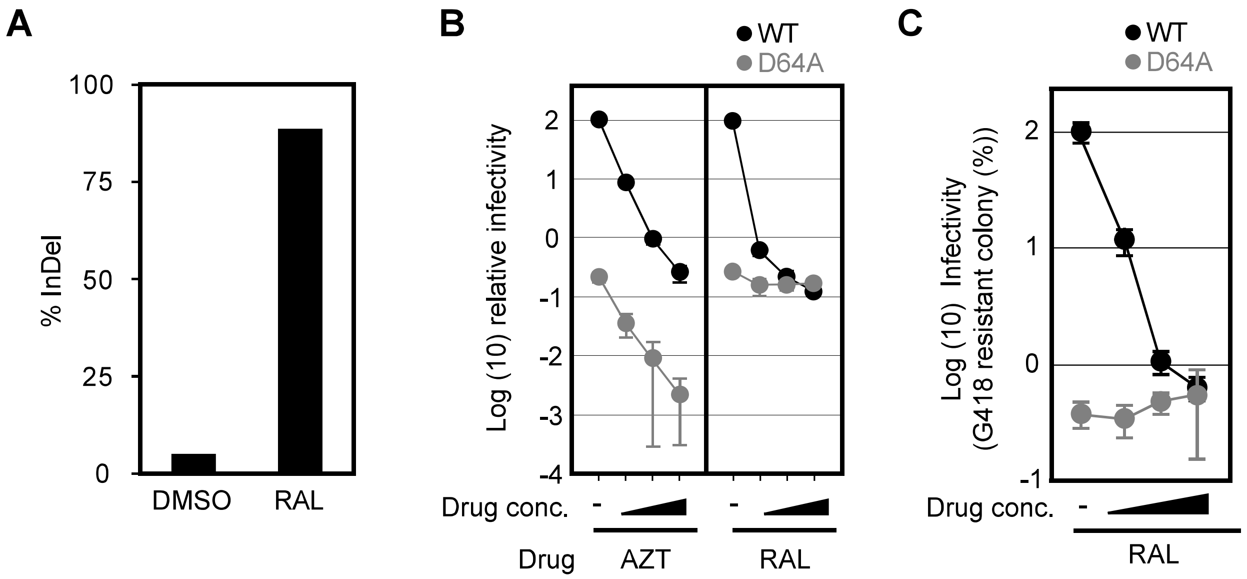

Additional File 1: Figure S5. The percentage of insertion and/or deletion (InDel) mutations at the host/viral junction. (**A**) LAM-PCR analysis. HT1080 cells were infected with VSVG-pseudotyped virus with Neo^R (NL-Neo-E(–)R(–)) in the presence or absence of 10 µM RAL. LAM-PCR was used to detect InDels at the host/viral junction. The individual sequence results were shown in Table 1. Effect of RAL on the infectivity of WT and D64A viruses by luciferase assay (**B**) and colony assay (**C**). (**B)** After infection with VSVG-pseudotyped WT (NL-Luc-E(–)R(–) or D64A (NL-Luc-IN-D64A-E(–)R(–)) virus in the presence of AZT or RAL, cells were harvested at 48 hpi and subjected to luciferase assay. Relative luciferase activity compared to a control sample, in which WT virus was infected without any compounds, were plotted. Concentration of AZT was 0, 1, 10 and 100 µM, whereas concentration of RAL was 0, 0.1, 1 and 10 µM. Black circles, WT virus; gray circles, D64A virus; ND, not detected. Error bars, s.d. of triplicate assays. (**C**) For colony formation assay, VSVG-pseudotyped WT (NL-Neo-E(–)R(–)) or D64A (NL-Neo-IN-D64A-E(–)R(–)) viruses, which had the neomycin resistant gene (NeoR), were used. HT1080 cells were continuously treated with 0, 0.1, 1, 10 µM RAL from -1 dpi. After selection with 600 µg/mL of G418, numbers of Neo^R colonies were counted. Error bars, s.d. of triplicate assays.

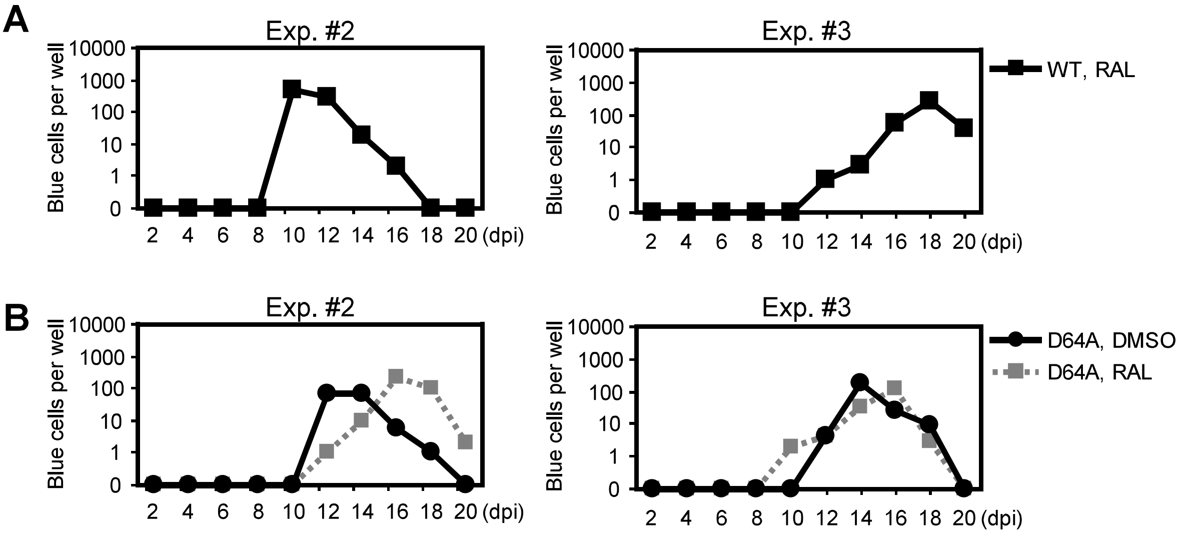

Additional File 1: Figure S6. Two additional independent data sets in Figure 5B and 5C. MT-4 cells were infected with replication competent WT virus (NL4-3), and the conditioned medium was harvested every 2 d. The infectivity of virions in the conditioned medium was evaluated using MAGIC5 cells, an indicator cell line (HeLa/CD4, CCR5, LTR-β-gal). MT-4 cells were infected with replication competent WT (**A**) or D64A (NL-IN-D64A) (**B**) virus in the presence or absence of RAL.

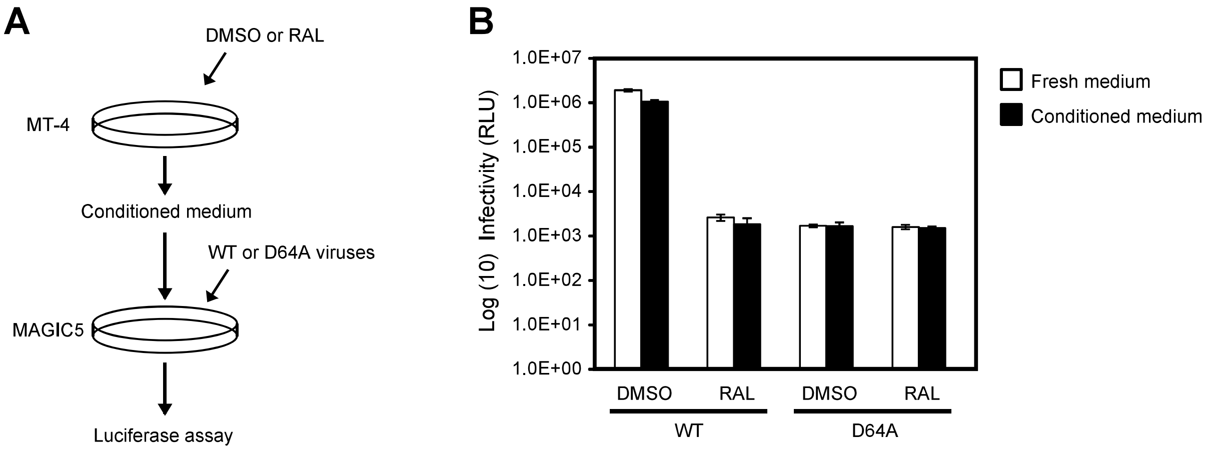

Additional File 1: Figure S7. RAL in 2d cultured conditioned medium is active. (**A**) Schematic outline of experimental design in (**B**). (**B**) MT-4 cells were cultured for 2d in medium supplemented with 10 µM RAL or DMSO. Conditioned medium (90 µL) and WT or D64A viruses (10 µL, 2 ng p24) were added to 1.0×10^4 MAGIC5 cells, then subjected to luciferase assay. For fresh medium treatment, RAL or DMSO were dissolved just before added to MAGIC5 cells.

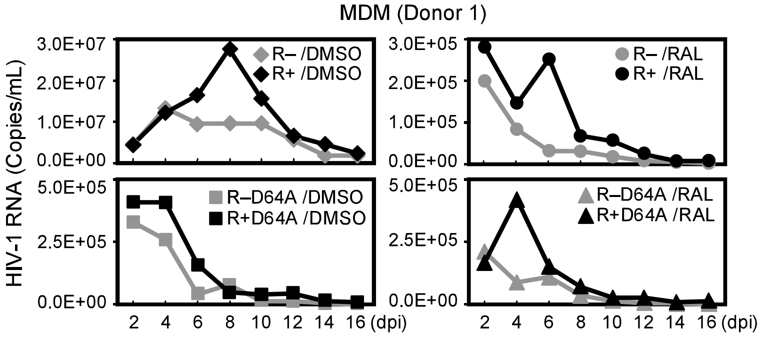

Additional File 1: Figure S8. Additional data sets with another donor in Figure 7D. HIV-1 replicates in MDMs in the presence of RAL. Replication competent NL4-3 with *env* derived from R5-tropic ADA viruses (NL-ADA, NL-ADA-R(–), NL-ADA-IN-D64A and NL-ADA-IN-D64A-R(–)) were infected, and HIV-1 RNA copy numbers in the conditioned medium were quantified by reverse transcription (RT)-qPCR.

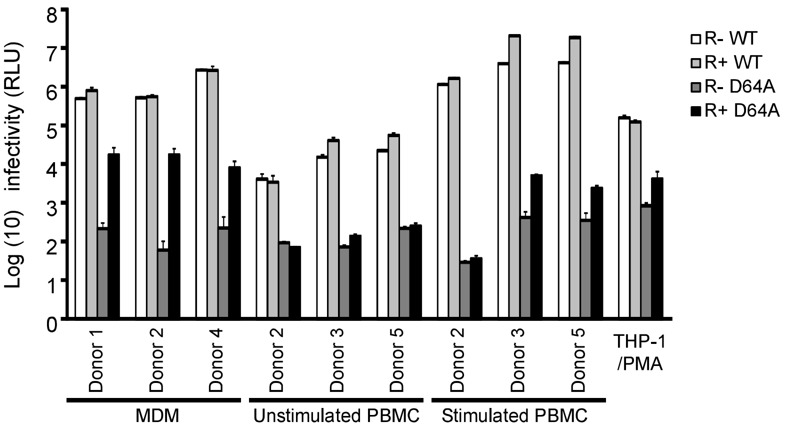

Additional File 1: Figure S9. Raw data in Figure 7E. Primary cells and cell lines were infected with IN WT or D64A mutant virus with or without Vpr. Cells were harvested at 48 hpi and cellular extracts were subjected to luciferase assay. White bars, WT/R–; light gray bars, WT/R+; dark gray bars, D64A/R–; black bars, D64A/R+.

Additional File 1: Table S1. RAL and EVG resistant mutations reported in literature.

IN mutations	IN inhibitors	References
N17S	RAL	30
P20K	EVG	29
H51Y	EVG	29
T66A/I/K	RAL/EVG	29-32
L68V/I	RAL/EVG	31
V72A/I	EVG	29-31
L74M	RAL/EVG	29-31
E92Q	RAL/EVG	29-32
Q95K	RAL/EVG	29, 31
T97A	RAL	30
F121Y	RAL/EVG	29, 31, 32
T124A	RAL/EVG	30
T125K	RAL/EVG	31
A128T	EVG	29, 30
E138A/K	RAL/EVG	29-32
G140A/S/C	RAL/EVG	29-32
Y143C/R/H	RAL	29, 30, 32
P145S	EVG	29, 30
Q146P/S/L	EVG	29-31
S147G	RAL/EVG	29-32
Q148K/R/H	RAL/EVG	29-32
V151I	RAL/EVG	30, 31
S153Y	RAL/EVG	29, 31, 32
M154I	RAL	30
N155H/S	RAL/EVG	29-32
E157Q	RAL/EVG	29, 31, 32
G163R	RAL	30
I204T	RAL	30
S230R	EVG	29
R263K	EVG	29, 31, 32

Additional File 1: Table S2. List of primers and probes used in this study.

Name	Sequence
Oligo cassette pair for the I-*Sce*I recognition DNA fragment to create pIRES2-EGFP-I-*Sce*I	
I-*Sce*I-sense	AATTCtagggataacagggtaatG
I-*Sce*I-antisense	GatccctattgtcccattaCCTAG
Primers to amplify the IRES fragment of pIRES2-EGFP-I-*Sce*I for the Southern blot probe	
pIRES2-EGFP+748F	TTCCACCATATTGCCGTCTTTTGG
pIRES2-EGFP+1172R	TCGACTAAACACATGTAAAGCATGTGC
Primers for I-*Sce*I-PCR (host DNA/5′-LTR junction) and I-*Sce*I-qPCR	
1st PCR	
pIRES2eGFP+543F	GGTGGGAGGTCTATATAAGCAGAG
pNL4-3+9207R	TTGTAGCACCATCCAAAGGTCAG
2nd PCR	
pIRES2eGFP+574F	AGTGAACCGTCAGATCCGCTAG
pNL4-3+98R+9173R	TGGTGTGTAGTTCTGCCAATCAG
Primers for I-*Sce*I-PCR (host DNA/3′-LTR junction)	
1st PCR	
pIRES2eGFP+1910R	CATGTGATCGCGCTTCT
L-M667	ATGCCACGTAAGCGAAACTCTGGCTAACTAGGGAACCCACTG
2nd PCR	
pIRES2eGFP+887R	GAGGAACTGCTTCCTTCACG
LambdaT	ATGCCACGTAAGCGAAACT
Primers to construct pIRES2-EGFP-5′-LTR to generate the HT-1080/pIRES2-EGFP-5′-LTR cell line as an I-*Sce*I-qPCR standard	
pNL4-3+9074F-*Sce*I-RI	gggaattctagggataaACTGGAAGGGCTAATTCACTCCCAAAG
pNL4-3+9423R-*Bam*HI	ggggatccTGTAGCAAGCTCGATGTCAGCAGTTC
Primers to amplify the rDNA fragment for the Southern blot probe	
rDNA+12089F	GAGTTTGACTGGGGCGGTACACC
rDNA+12390R	GGCGAATTCTGCTTCACAATGATAGG
Primers to amplify the I-*Ppo*I cDNA for Ad-I-*Ppo*I vector construction	
Adeno-*Ppo*I-DraI-F	TTTAAAcaccatggactacaaagacgatgacgacaagGATCCAAAAAAGAAGA GAAAGGTAatggcgctcaccaatgctcaaatc
Adeno-*Ppo*I-DraI-R	TTTAAAGATATCttataccacaaagtgactgcccctttg
Primers and TaqMan probes for quantify the HIV-1 DNA	
Early RT	
M667	GGCTAACTAGGGAACCCACTGC
AA55	CTGCTAGAGATTTTCCACACTGAC
R/U5 (probe)	TAGTGTGTGCCCGTCTGTTGTGTGAC
Late RT	
M667	GGCTAACTAGGGAACCCACTGC
M661	CCTGCGTCGAGAGATCTCCTCTG
R/U5 (probe)	TAGTGTGTGCCCGTCTGTTGTGTGAC
2-LTR	
MH535	AACTAGGGAACCCACTGCTTAAG
2-LTR-AS	TGGTGTGTAGTTCTGCCAATCA
U3 (probe)	CCTTGATCTGTGGATCTACCACACACAAGGC
Alu-PCR (1st round)	
first-Alu-F	AGCCTCCCGAGTAGCTGGGA
first-Alu-R	TTACAGGCATGAGCCACCG
first-gag-R	CAATATCATACGCCGAGAGTGCgcgcttcagcaag
Alu-PCR (2nd round)	
second-tag-R	CAATATCATACGCCGAGAGTGC
2-LTR-S	CCCTCAGACCCTTTTAGTCAGTG
probe-2 (probe)	CGCTTCAGCAAGCCGAGTCCTGC
β-globin	
globin-F	ACACAACTGTGTTCACTAGC
globin-R	CAACTTCATCCACGTTCACC

Additional File 1: Table S2. (Continued) List of primers and probes used in this study.

Name	Sequence
Primers for I-*Ppo*I-PCR (direct repeat orientation)	
1st PCR	
rDNA+11784R	TGGCTGTGGTTTCGCTGGATAGTAG
pLenti6+5208F	TGGAAGGGCTAATTCACTCCC
2nd PCR	
rDNA+11747R	GGAATCTCGTTCATCCATTCATGC
pLenti6+5232F	CAAGATCTGCTTTTTGCTTGTACTG
Primers for I-*Ppo*I-PCR (inverted repeat orientation)	
1st PCR	
rDNA+11589F	GTGTTGACGCGATGTGATTTCTGC
pLenti6+5208F	TGGAAGGGCTAATTCACTCCC
2nd PCR	
rDNA+11612F	CCCAGTGCTCTGAATGTCAAAGTGAAG
pLenti6+5232F	CAAGATCTGCTTTTTGCTTGTACTG
Primers and TaqMan probe for I-*Ppo*I-qPCR (direct repeat orientation)	
rDNA+11725R	GCGCGTCACTAATTAGATGACG
pLenti6+5237F	CAAGATCTGCTTTTTGCTTGTACTG
pLenti6-LTR (probe)	TGCTTCAAGTAGTGTGTGCCCGTCTGTTGT
Primers and TaqMan probe for I-*Ppo*I-qPCR (inverted repeat orientation)	
rDNA+11645F	AATGAAGCGCGGGTAAACG
pLenti6+5237F	CAAGATCTGCTTTTTGCTTGTACTG
pLenti6-LTR (probe)	TGCTTCAAGTAGTGTGTGCCCGTCTGTTGT
Primers and TaqMan probe for EGFP-qPCR	
EGFP-F	GAAGAACGGCATCAAGGTGAA
EGFP-R	ACTGGGTGCTCAGGTAGTGGTT
EGFP-probe (probe)	TCAAGATCCGCCACAACATCGAGGA
Primers for LAM-PCR	
Adapter	
LC-Long	GACCCGGGAGATCTGAATTCAGTGGCACAGCAGTTAGG
LC-MseI	TACCTAACTGCTGTGCCACTGAATTCAGATC
Linear PCR primer	
pNL4-3+9264R-Biotin	Biotin-CTCCTTTATTGGCCTCTTCTACCTTATC
1st exponential PCR primers	
LCI	GACCCGGGAGATCTGAATTC
pNL4-3+104R/+9179R-Biotin	Biotin-TGGCCCTGGTGTGTAGTTCTG
2nd exponential PCR primers	
LCII	AGTGGCACAGCAGTTAGG
pNL4-3+75R/+9150R	GGAAGTAGCCTTGTGTGTGGTAGATC
Primers to amplify IN fragment of HIV-1	
pNL+4207F	GTGCTGGAATCAGGAAAGTAC
pNL+5120R	TGTTTTACTAATCTTTTCCATGTG

Supplementary Methods

Southern blot analysis

To estimate I-*Sce*I site cleavage efficiency, THP-1/I-*Sce*I cells were differentiated into monocytoid cells with PMA and then infected with the adenovirus vectors Ad-I-*Sce*I or Ad-LacZ for 1 h. These cells were harvested at 12 h post-infection (hpi), and genomic DNA was digested using *Nco*I. Southern blot analysis was carried out using a digoxigenin (DIG) system (Roche Diagnostics) according to the manufacturer's instructions. To prepare the hybridization probe, a DNA fragment encoding IRES was amplified from pIRES2-EGFP-I-SceI by PCR with the primers pIRES2-EGFP+748F and pIRES2-EGFP+1172R (Additional File 1: Table S2) using a PCR DIG Probe Synthesis kit (Roche Diagnostics). To estimate I-*Ppo*I site cleavage efficiency, 4-hydroxytamoxifen (4-OHT) was added to the medium of HEK293T/ER-I-*Ppo*I and HEK293T/Vector for 24 h to a final concentration of 1 μM to induce ER-I-*Ppo*I expression. The cells were harvested, and genomic DNA was digested using *Nco*I. As a positive control, *Nco*I-digested HEK293T cell genomic DNA was further digested with I-*Ppo*I (Promega). Southern blot analysis was carried out using a DIG system. To prepare the hybridization probe, an rDNA fragment was amplified by PCR with the primers rDNA+12089F and rDNA+12390R (Additional File 1: Table S2) using a PCR DIG Probe Synthesis kit.
